# Natural Coumarin Isomers with Dramatically Different AIE Properties: Mechanism and Application

**DOI:** 10.1021/acscentsci.3c00012

**Published:** 2023-04-19

**Authors:** Shan-Shan Chen, Haoran Wang, Bo Wu, Qiyao Li, Junyi Gong, Yun-Li Zhao, Yun Zhao, Xia Xiao, Jacky W. Y. Lam, Zheng Zhao, Xiao-Dong Luo, Ben Zhong Tang

**Affiliations:** † State Key Laboratory of Phytochemistry and Plant Resources in West China, Kunming Institute of Botany, Chinese Academy of Sciences, Kunming 650201, PR China; ‡ School of Science and Engineering, Shenzhen Institute of Aggregate Science and Technology, The Chinese University of Hong Kong, Shenzhen, Guangdong 518172, China; § Key Laboratory of Medicinal Chemistry for Natural Resource, Ministry of Education and Yunnan Province, Yunnan Characteristic Plant Extraction Laboratory, School of Chemical Science and Technology, Yunnan University, Kunming 650500, PR China; ∥ Hong Kong Branch of Chinese National Engineering Research Center for Tissue Restoration and Reconstruction and Department of Chemistry, The Hong Kong University of Science and Technology, Clear Water Bay, Kowloon, Hong Kong, China; ⊥ University of Chinese Academy of Sciences, Beijing 100049, PR China

## Abstract

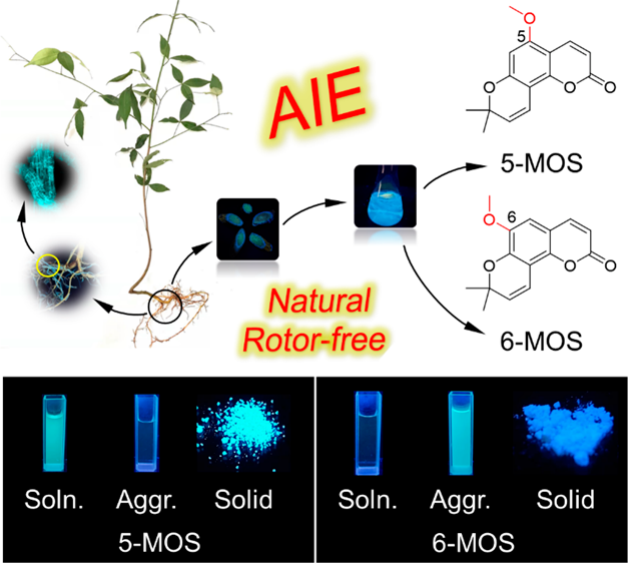

Aggregation-induced emission luminogens (AIEgens) are of great importance in optoelectronics and biomedical fields. However, the popular design philosophy by combining rotors with traditional fluorophores limits the imagination and structural diversity of AIEgens. Inspired by the fluorescent roots of the medicinal plant *Toddalia asiatica*, we discovered two unconventional rotor-free AIEgens, 5-methoxyseselin (5-MOS) and 6-methoxyseselin (6-MOS). Interestingly, a slight structural difference of the coumarin isomers leads to completely contrary fluorescent properties upon aggregation in aqueous media. Further mechanism investigation indicates that 5-MOS forms different extents of aggregates with the assistance of protonic solvents, leading to electron/energy transfer, which is responsible for its unique AIE feature, i.e., reduced emission in aqueous media but enhanced emission in crystal. Meanwhile, for 6-MOS, the conventional restriction of the intramolecular motion (RIM) mechanism is responsible for its AIE feature. More interestingly, the unique water-sensitive fluorescence property of 5-MOS enables its successful application for wash-free mitochondria imaging. This work not only demonstrates an ingenious tactic to seek new AIEgens from natural fluorescent species but also benefits the structure design and application exploration of next-generation AIEgens.

## Abstract 2

Inspired by the fluorescent roots of a medicinal plant, we discovered a pair of natural AIE molecules. The molecules look similar but show distinct fluorescent properties when aggregating in water.

## Introduction

Over the long course of evolution, nature has optimized sophisticated materials and systems with diverse functions. Numerous great inventions throughout human history are inspired by nature and play indispensable roles in human life. With the rapid advancement of technology, scientists tend to cognize nature from the microscopic level, thus motivating the blossom of functional materials and systems, such as 3D-microfliers inspired by wind-dispersed seeds, soft robotics inspired by soft-bodied animals, and self-illuminous coating inspired by the lotus leaf.^1−5^ Most strikingly, the exploration tempted by fascinating luminous jellyfish led to the discovery of green fluorescent protein (GFP), which has been widely used as a fluorescent tag in living organisms and revolutionized analytical technology in biological research.^6^ Further studies have revealed that the fluorescence of GFP arises from an internal *p*-hydroxybenzylideneimidazolidinone chromophore, and then, many analogues have been tailored with improved fluorescence properties and excellent functions.^7−10^ In addition to luminous jellyfish, plants produce abundant natural fluorescent compounds with varied biological and pharmacological activities, but their fluorescent characteristics are rarely investigated and exploited.^11^ These endogenous fluorophores in plants will be an incredible source of inspiration for developing novel structural fluorescent materials.

Organic fluorescent materials have made outstanding achievements in visualization.^12−14^ However, the traditional organic fluorescent molecules frequently suffer from the fluorescence quenching effect in the solid or aggregate state, restricting their practical applications in optoelectronics or biological systems. Fortunately, some molecules were discovered with an aggregation-induced emission (AIE) property, and they can exhibit strong emission in the aggregate state.^15−19^ The generally accepted working mechanism of AIE is the restriction of intramolecular motion (RIM); later, detailed investigations indicated that intramolecular motion mostly induced molecular conformation change, which is strongly correlated with many nonradiative decay pathways like vibronic coupling, conical intersection, and photochemical reactions, etc.^20−26^ Currently, it has become an effective and reliable method to develop AIE luminogens (AIEgens) through linking propeller-like moieties or rotors with traditional fluorophores based on the RIM mechanism.^27−32^ A large amount of AIEgens have been designed and synthesized and widely used in optical devices, luminescent sensors, bioimaging, and theranostics.^33−40^ However, the popular design principle constrains the imagination of structural diversity to some extent. Besides, these artificially synthesized AIEgens commonly have the shortcomings of complex synthesis, environmental toxicity, and poor biocompatibility. Thus, it is urgently needed to think outside the box and further expand AIEgen systems.^41^

Natural products are endowed with inconceivable structural skeletons and good biocompatibility. Their unique and diverse structural skeletons have enriched the imagination space of new structure design of functional materials including luminescent materials.^42−44^ Coumarins are a class of fluorescent natural products containing a 2*H*-chromen-2-one motif, abundant in many plants. In addition to their diverse pharmacological activities, they have excellent optical properties and been widely used as fluorescent chemosensors.^45^ However, scarcely any natural coumarins were reported to be AIE-active because of the π-conjugated planar skeletons.^46^ Hence, the exploration of natural AIE coumarins is extremely important for fundamental understanding of planar AIE systems. Moreover, it has been reported that isomers with minimal structural dissimilarities can exhibit significant property variations.^47−51^ For example, the varied conjugation effect and steric hindrance caused by the structural isomerism will influence the room-temperature phosphorescence (RTP) performance.^52^ Therefore, isomers could be ideal molecular models utilized for systematically investigating the structure–property relationships of luminogens. Marvelously, natural plants are adept at creating isomeric molecules, due to their various biosynthetic enzymes, which is a technologically tough task for organic synthesis. In this context, it is very desirable and significant to seek isomeric AIE coumarins from fluorescent plants, and the comprehensive understanding of their working mechanism will in turn guide the rational design of novel AIE materials.

Herein, we reported two AIE coumarin isomers, 5-methoxyseselin (5-MOS) and 6-methoxyseselin (braylin, 6-MOS), isolated from the fluorescent roots of *Toddalia asiatica*, a traditional folk medicine mainly used for curing rheumatic arthritis and traumatic injury, etc.^53^ To the best of our knowledge, this is the first time that natural coumarins without artificial structural modification are reported to be AIE-active. By photophysical measurements, single-crystal structure analysis, and DFT calculations, we found that 6-MOS showed aggregation-enhanced emission (AEE) properties due to the normal RIM mechanism. However, an abnormal decreased emission was observed when adding water into 5-MOS solution to form aggregates, which is contrary to the behavior of traditional AIEgens. Further exploration suggested that different assemblies or aggregates may form upon the addition of protonic solvents, and the energy/electron transfer within the different extents of 5-MOS aggregates may contribute to quench the exciton, leading to the decreased emission in aqueous media. However, bright emission could be achieved in crystal, in which only long-range ordered assembly is formed, which avoids the occurrence of energy/electron transfer. More interestingly, the dim fluorescence of 5-MOS in aqueous media could also be switched on when entering cellular mitochondria, which will enable its novel application in wash-free mitochondria imaging and also help the understanding of molecular behavior of dyes in cells.

## Results and Discussion

### Discovery of Natural AIE Coumarins

**Figure 1:**
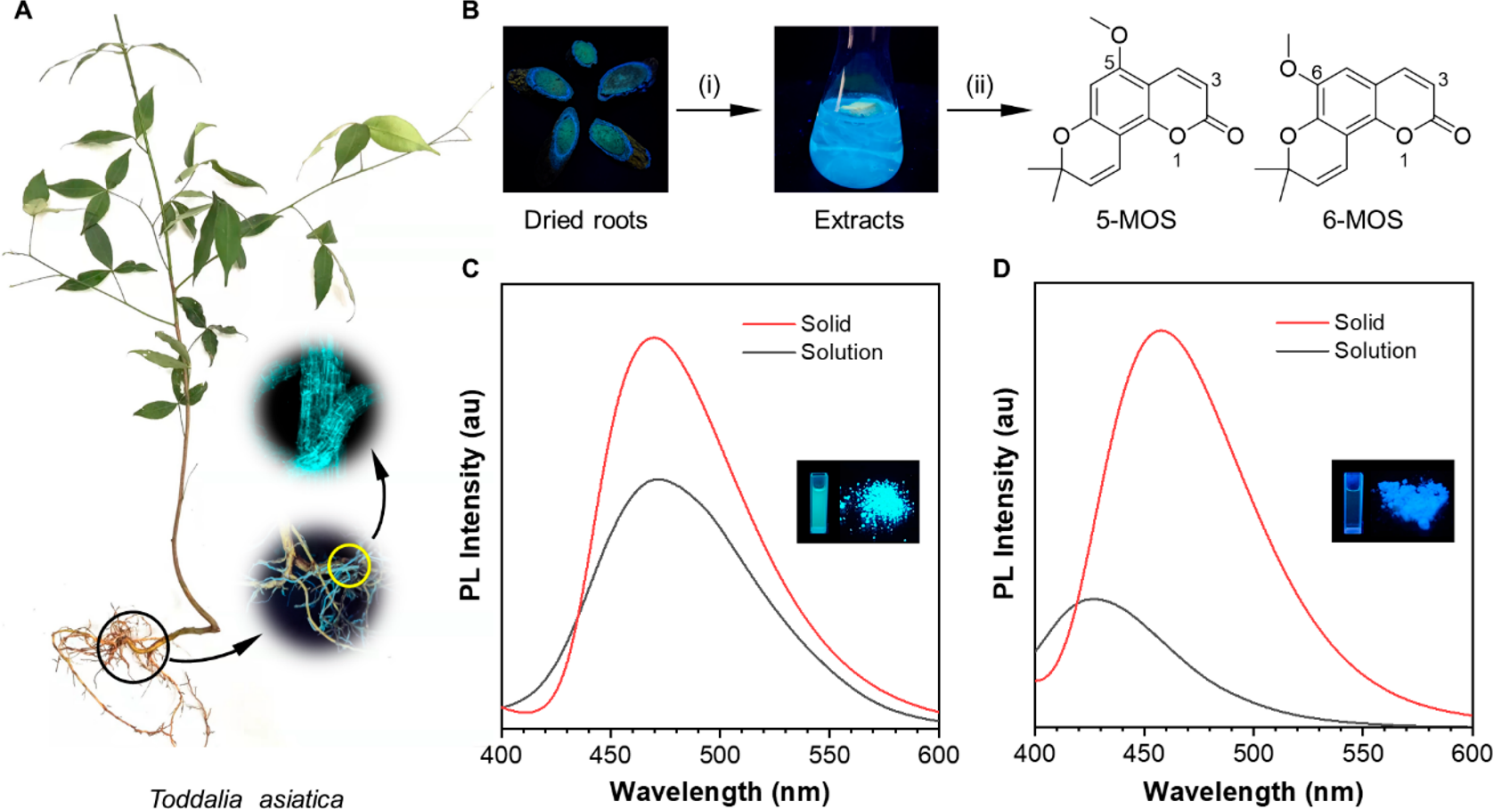
Discovery of natural AIE coumarins. (A) The fresh plant of *Toddalia asiatica* and its fluorescent roots taken under 365 nm UV light irradiation and confocal microscope. (B) Preparation process and chemical structures of 5-MOS and 6-MOS: (i) extraction; (ii) isolation. (C,D) PL spectra of 5-MOS (C) and 6-MOS (D) in solution (DMSO) and solid state. Inset: Fluorescence images taken under 365 nm UV light irradiation. Concentration: 10 μM. Excitation wavelength: 328 nm for 5-MOS and 352 nm for 6-MOS.

*T. asiatica* is a medicinal plant widely distributed in southern Asia and eastern Africa that contains diversified coumarins and alkaloids with broad pharmacological activities.^53,54^ We coincidently noticed its dried roots could display bright blue fluorescence under 365 nm UV light irradiation (Figure 1A). Driven by curiosity, we observed the roots of fresh plant under UV light and confocal fluorescence microscope, respectively, and the tender roots of *T. asiatica* were also emissive. These phenomena implied some AIE molecules may exist in this plant. After a series of extraction and isolation procedures, 5-MOS and 6-MOS, a pair of coumarin isomers, were obtained (Figures 1B, S1–7). They have similar structures with a small difference in the substituent position of the methoxy group; however, discrepant fluorescence properties have been exhibited. As shown in Figure 1C,D, 5-MOS emitted brightly in DMSO solution with an emission maxmium of 473 nm, while the fluorescence emission of 6-MOS solution was very weak with an emission maxmium of 428 nm. The fluorescence quantum yields of 5-MOS and 6-MOS in DMSO solution were 11.8 and 2.1%, and the average fluorescence lifetimes of them were 3.09 and 0.62 ns, respectively (Table S1 and Figure S8). Meanwhile, both compounds showed enhanced emission in the solid state compared to those in the solution state, and the emission wavelength of 5-MOS in the solid state is almost unchanged, while that of 6-MOS exhibits an obvious redshift. The fluorescence quantum yield and the average fluorescence lifetime of 5-MOS powders were 18.5% and 5.53 ns, while those of 6-MOS in the solid state were 6.5% and 3.13 ns. These data implied both the two pyranocoumarins are AEE-active.

### Single-Crystal Structure Analysis and Theoretical Calculation

**Figure 2:**
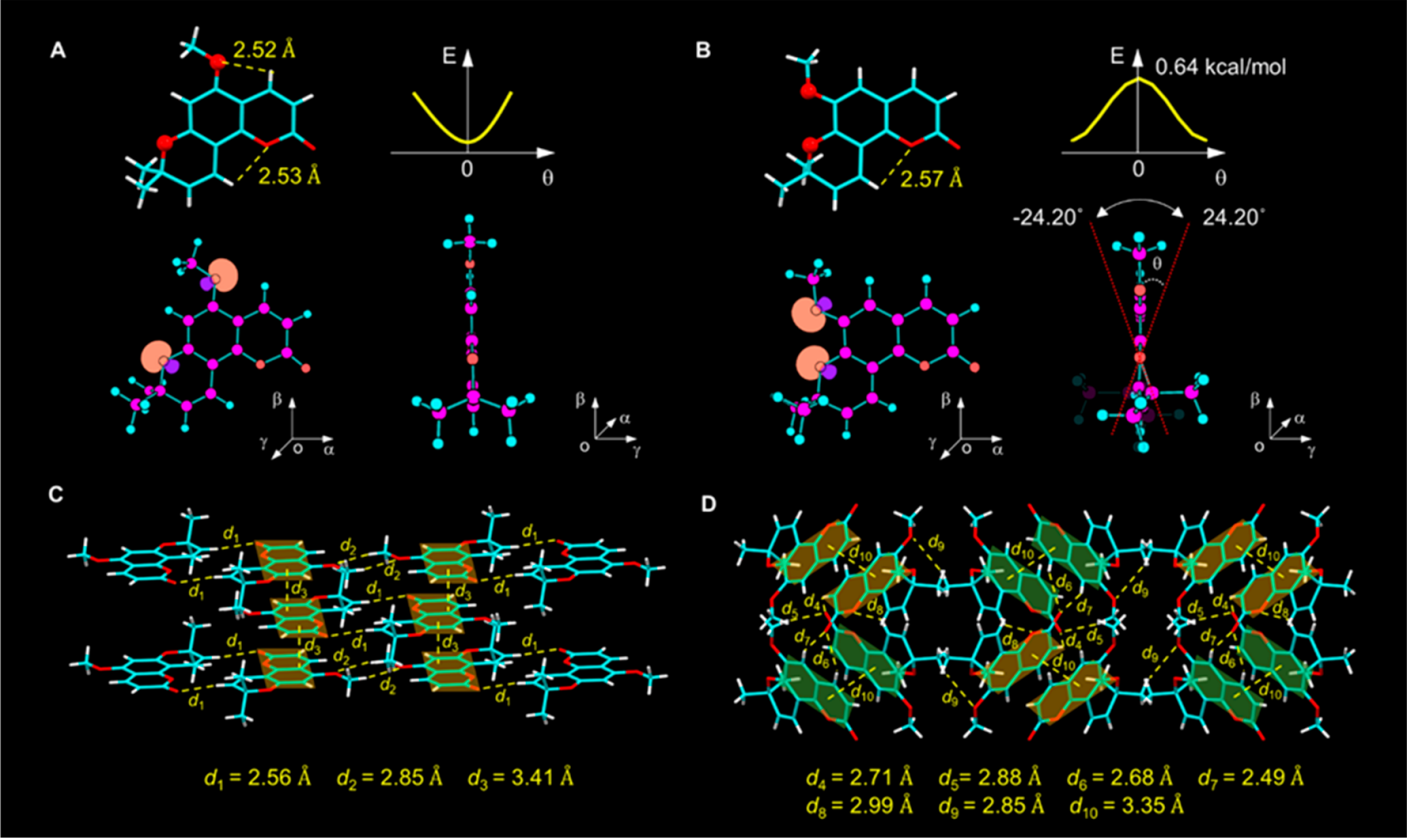
Single-crystal structure analysis and theoretical calculation. (A,B) Crystal structures, optimized conformations with natural bond orbital of labeled oxygen atoms, and potential energy surface of excited state torsion of 5-MOS (A) and 6-MOS (B). (C,D) Single-crystal packing structure and intermolecular noncovalent interactions of 5-MOS (C) and 6-MOS (D).

To gain an insight into the mechanism behind their AIE behavior, the single-crystal structures of the two isomers were obtained and analyzed. For 5-MOS, it displays a planar configuration and intramolecular C–H···O interactions with the distances of 2.52 and 2.53 Å (Figures 2A and S9A). The optimized conformation of 5-MOS by the theoretical calculation shows that the natural bond orbitals of the labeled oxygen atoms will not influence each other, and the potential energy surface of the excited state torsion of 5-MOS reveals that the planar conformation with the lowest energy is thermodynamically stable (Figure 2A). Thus, the planar and rigid conformation of 5-MOS matched well with its strong emission in the solution state. Besides, the fine absorption peaks of 5-MOS in DMSO also reflect the structural rigidity of 5-MOS, in line with the analysis above (Figure S7). In the crystal of 5-MOS, the molecules align regularly into a layered structure in an antiparallel displaced manner with an interlayer distance of 3.41 Å, and some C–H···O interactions with distances of 2.56 and 2.85 Å can be observed between adjacent molecules (Figures 2C and S10). For 6-MOS, it adopts a nonplanar conformation with a dihedral angle of 27.35° between the pyranoid ring and the benzo-α-pyranone parent nucleus, and the oxygen atoms of the 6-methoxy group and pyranoid ring are slightly deviated from the parent nucleus plane, suggesting that the rigidity of the molecular structure is kind of weakened, and intramolecular vibration/twisting may occur upon photoexcitation (Figure S9B–C). The intramolecular C–H···O interaction with the distance of 2.57 Å is weaker than those of 5-MOS, and the motion of the methoxy group at position-6 is not restricted (Figure 2B). The optimized conformation of 6-MOS by the theoretical calculation shows that the natural bond orbitals of the two labeled oxygen atoms can repel each other. Furthermore, the potential energy surface of excited state torsion indicates that the twisted conformations are more stable, and active excited state vibration is favorable (Figure 2B and Video S1). The single-crystal structure analysis and theoretical calculation results suggest that the twisted conformation of 6-MOS can easily undergo active molecular backbone vibration or twisting, which contribute to the nonradiative decay and bring about the dim emission of 6-MOS in DMSO. While in crystal of 6-MOS, as shown in Figures 2D and S10, the pairwise antiparallel displaced dimers can be observed obviously with the interplanar distance of 3.35 Å, and every dimer is spatially staggered with the neighboring ones to assemble into a 3D network-stacking structure. Multiple intermolecular C–H···O interactions with distances ranging from 2.49 to 2.99 Å among these molecules could effectively restrict the intramolecular vibrations and make 6-MOS emissive strongly in solid state compared to the solution state.

### Abnormal Phenomenon of AIE

**Figure 3:**
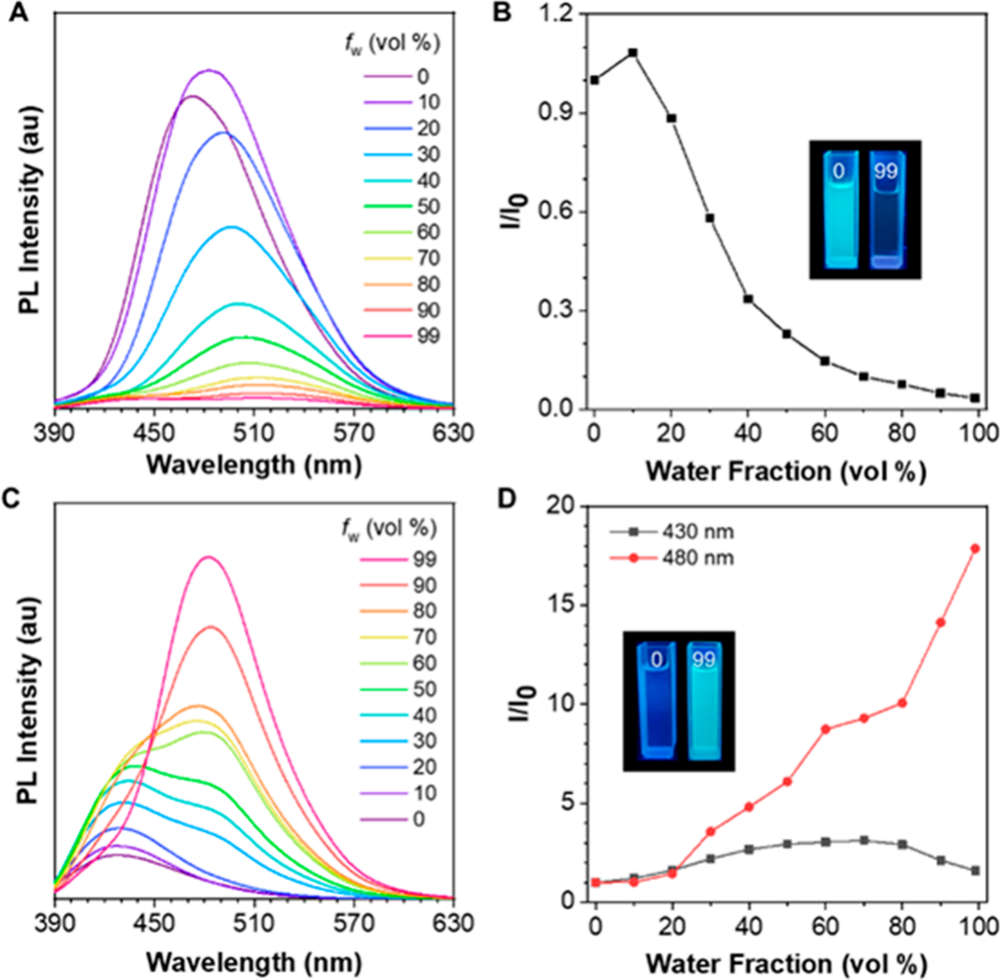
AIE curves of 5-MOS and 6-MOS. (A,C) PL spectra of 5-MOS (A) and 6-MOS (C) in DMSO/water mixtures with different water fractions (*f*_w_). Concentration: 10 μM. Excitation wavelength: 328 nm for 5-MOS and 352 nm for 6-MOS. (B,D) Plots of the relative emission intensity (*I*/*I*_0_) versus *f*_w_ of 5-MOS (B) and 6-MOS (D), where *I* and *I*_0_ are the maximal PL intensity in DMSO/water mixtures with different *f*_w_ and in DMSO solution, respectively. Inset: Fluorescence images at *f*_w_ = 0% and *f*_w_ = 99% taken under 365 nm UV light irradiation.

To further explore their luminescence properties in the aggregate state, PL spectra of the two coumarin isomers in different DMSO/water mixtures were measured. Surprisingly, for 5-MOS, with the progressively increased water fraction (*f*_w_), the emission wavelength slightly redshifted, while the emission intensity reduced gradually, and it was nearly nonemissive when the *f*_w_ was increased up to 99%, seemingly like an aggregation-caused quenching (ACQ) molecule (Figure 3A,B). On the contrary, 6-MOS exhibited enhanced emission with increasing *f*_w_, and a new emission peak of aggregates at 480 nm was observed clearly, which could be ascribed to the emission of aggregates (Figure 3C,D). The abnormal result of 5-MOS challenged our perception of the AIE molecule, so another two solvent systems, ethanol/water and THF/water, were chosen to carry out the same test. As shown in Figures S11 and S12, similar results were observed except for an interesting difference of 5-MOS in the THF/water mixture, which first showed weak emission in pure THF but turned to strong emission with *f*_w_ at 10%, and then, the fluorescence attenuated gradually with *f*_w_ increased from 10 to 99%. Dynamic light scattering (DLS) results indicated that aggregates were indeed formed in the poor solvent water (Figure S13). The abnormal AIE curves of 5-MOS stimulated further exploration of the concentration effect, and the result showed that although the emission intensity gradually increased with the concentration due to the formation of aggregates, both the absorption and emission wavelength barely changed. This result could exclude the formation H-aggregation,^55^ which is characterized by a large extent of absorption blueshift and has been well-known as a quenching factor of fluorescence (Figures S14 and S15). Moreover, the gradually enhanced fluorescence intensity with concentration and the brightly emissive crystals of 5-MOS further confirm the absence of H-aggregation but validate the AEE character, since H-aggregation mostly leads to the quench of the emission. Then, why does the AIE molecule 5-MOS exhibit an ACQ-like property and quench its fluorescence in mixed solvents with a high content of water? We deduced that the solvent may play an important role in quenching the emission of 5-MOS.

### Solvent Effects and Proposed Mechanism Diagram

**Figure 4:**
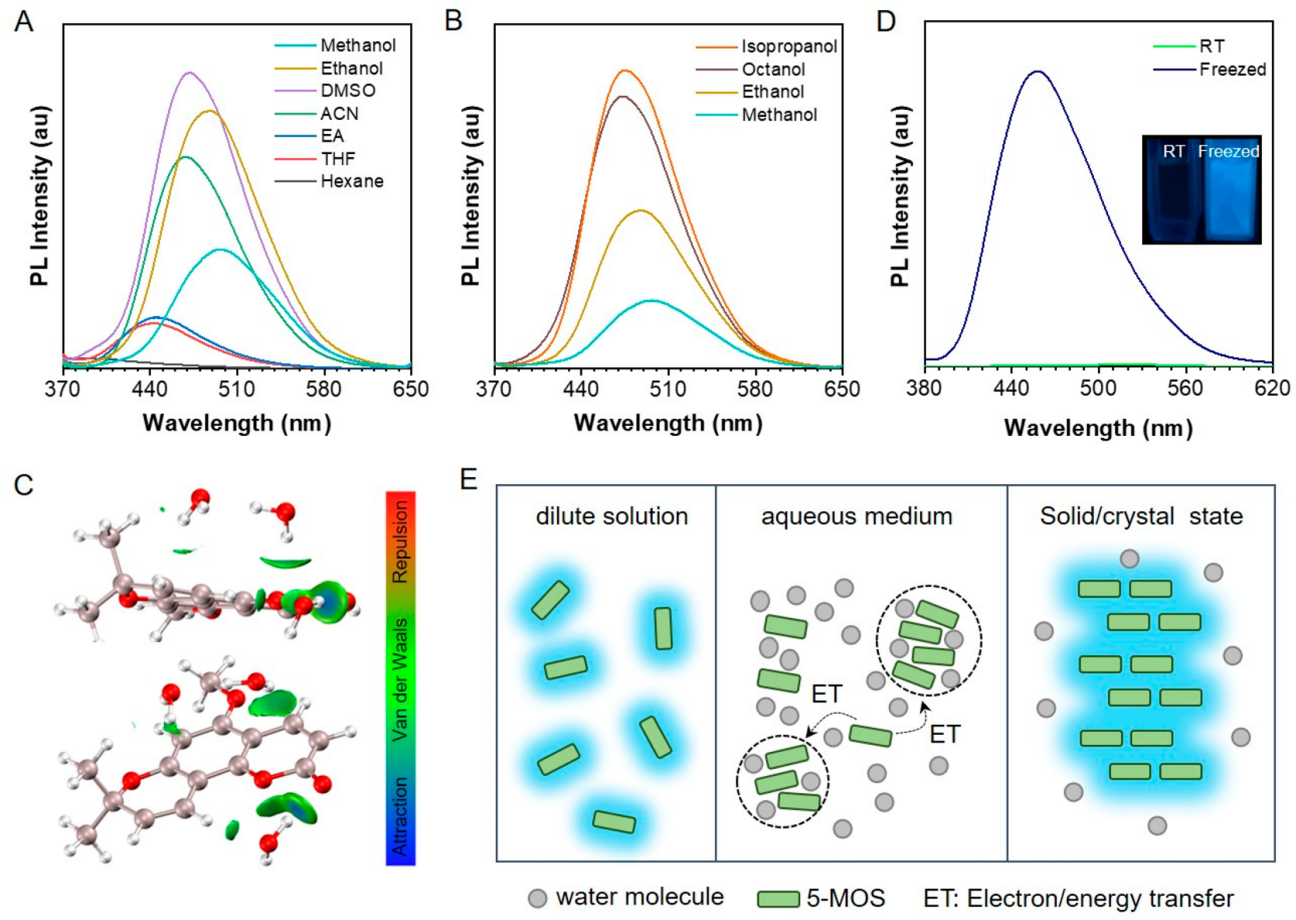
Solvent effects and proposed schematic diagram of mechanism of 5-MOS. (A) PL spectra of 5-MOS in solvents with different polarities. (B) PL spectra of 5-MOS in different protic solvents. (C) Intermolecular interaction between 5-MOS and water molecules in excited state calculated via independent gradient model based on Hirshfeld partition (IGMH). δg_inter isovalue = 0.0005. (D) PL spectra of 5-MOS in ethanol/water mixtures with *f*_w_ = 99%. Inset: Fluorescence images of 5-MOS in ethanol/water mixtures with *f*_w_ = 99% taken under 365 nm UV light irradiation. Concentration: 10 μM. Excitation wavelength: 328 nm. (E) Schematic illustration of 5-MOS in dilute solution, aqueous medium, and in the solid state.

To prove the above conjecture, the PL performance of 5-MOS in a series of solvents with different polarities was tested. As shown in Figure 4A, from hexane to DMSO, with the increase of solvent polarity, the PL intensity of 5-MOS increased gradually with a pronounced redshift of maximum emission wavelength from 404 to 470 nm. This might be attributed to the suppression of the proximity effect (SOPE), which commonly existed in many aromatic carbonyl and nitrogen heterocyclic compounds.^56,57^ However, the fluorescence intensity did not increase all the way; instead, it decreased gradually from ethanol to methanol. More specifically, the stronger the hydrogen-donating capability and smaller the size of the solvent molecules, the weaker the fluorescence of 5-MOS, which suggested protic solvents might be crucial to the fluorescence quenching of 5-MOS aggregates (Figure 4B). The phenomenon of protic solvents quenching emission has also been observed by Han and co-workers, and with the support of experimental data, they assigned the reduced emission to the formation of the intermolecular hydrogen bond.^58−60^ To further unveil whether our system has a relationship with hydrogen bond formation or not, an independent gradient model based on Hirshfeld partition (IGMH) was employed to visually present intermolecular interactions between 5-MOS and water molecules in an aqueous environment. The results showed that both in the ground and excited state, 5-MOS was surrounded by water molecules through Van der Waals interactions and/or H-bonds between the hydrogen of water and carbonyl group of coumarin (Figures 4C and S16). Furthermore, we also implemented in situ IR measurement, which indicated that at a *f*_w_ with an obvious fluorescence intensity decrease, the wavenumber of the carbonyl group showed an obvious blueshift, which is in accordance with reference and suggests the formation of intermolecular H-bonds with water (Figure S17).^60^ It is worthy to note that the quenched emission of 5-MOS could recover upon freezing at 77 K (Figure 4D). Based on these experimental data, we proposed a possible mechanism to explain the fluorescence phenomenon of 5-MOS as follows (Figure 4E). In general, when 5-MOS molecules dissolved in good solvents, bright emission was observed due to the rigid conformation of 5-MOS. Upon adding water, however, 5-MOS will randomly aggregate, and the water molecules will insert into 5-MOS aggregates through an intermolecular H-bond interaction, leading to the formation of different extents of aggregates. Then, electron/energy transfer may occur among 5-MOS molecules and the different extents of aggregates upon photoirradiation, which would quench the emission.^61,62^ When freezing the aggregates’ aqueous mixture (Figure 4E) by liquid nitrogen, the water molecules inserted into the aggregates can be squeezed out during the crystallization process of 5-MOS, and the aggregates of 5-MOS in the mixed solvents may further reassemble to form larger uniform aggregates or microcrystals,^63^ which could block the electron/energy transfer, thus affording strong emission. Also, the emission peak at 462 nm after freezing was close to the emission of 5-MOS crystals, further supporting our conclusion. It is worthy to note that this conclusion also matched well with the emission of 5-MOS in different alcohols (Figure 4B), in which smaller size alcohols with stronger H-bond formation capability exhibited the weakest emission. In addition, the solvent effects of 6-MOS were also analyzed, and the results indicated that the PL intensity could only be influenced by the solvent polarity but was nearly unaffected by the protic solvents (Figure S18). Thus, the enhanced fluorescence of 6-MOS in water could mainly be attributed to the conventional mechanism of RIM.

### Wash-Free Cell Imaging

**Figure 5:**
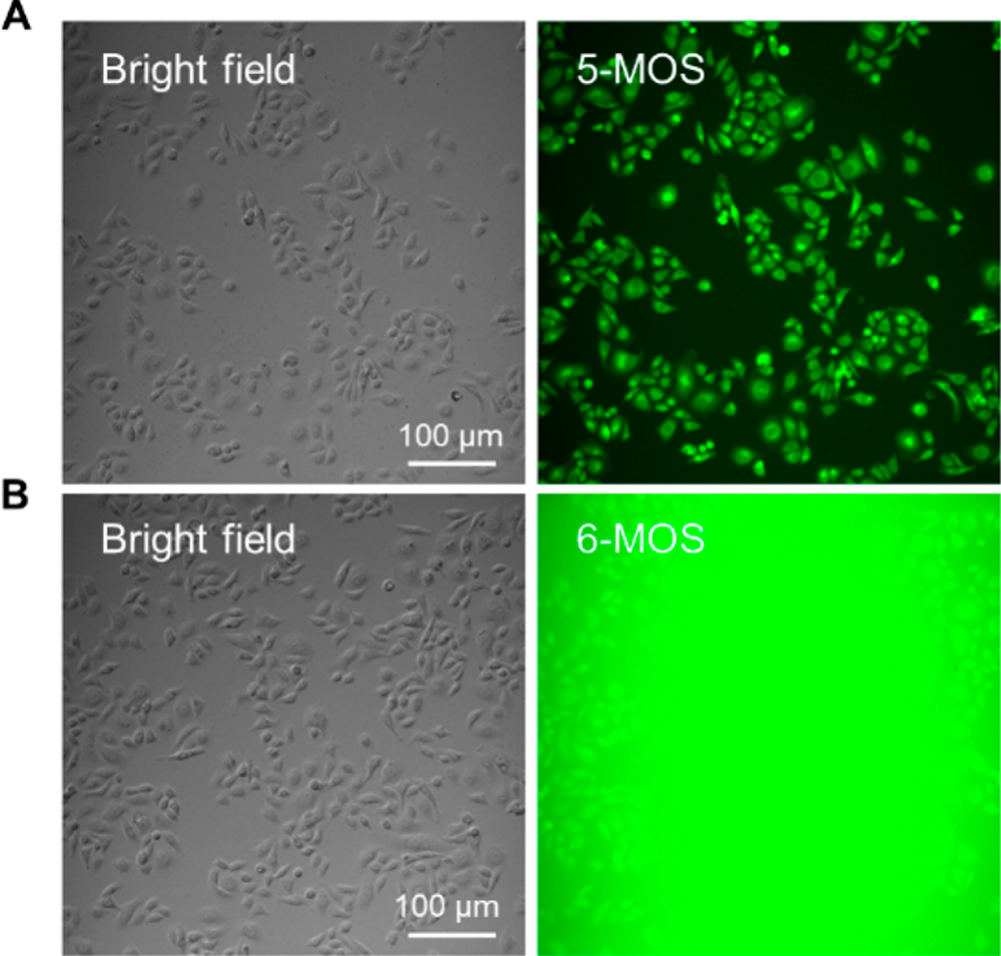
Wash-free cell imaging of 5-MOS. (A) Images of MHCC97H cells stained with 5-MOS for 15 min without the washing process taken by fluorescence microscope. (B) Images of MHCC97H cells stained with 6-MOS for 1.5 h without the washing process taken by fluorescence microscope. Concentration: 10 μM.

**Figure 6:**
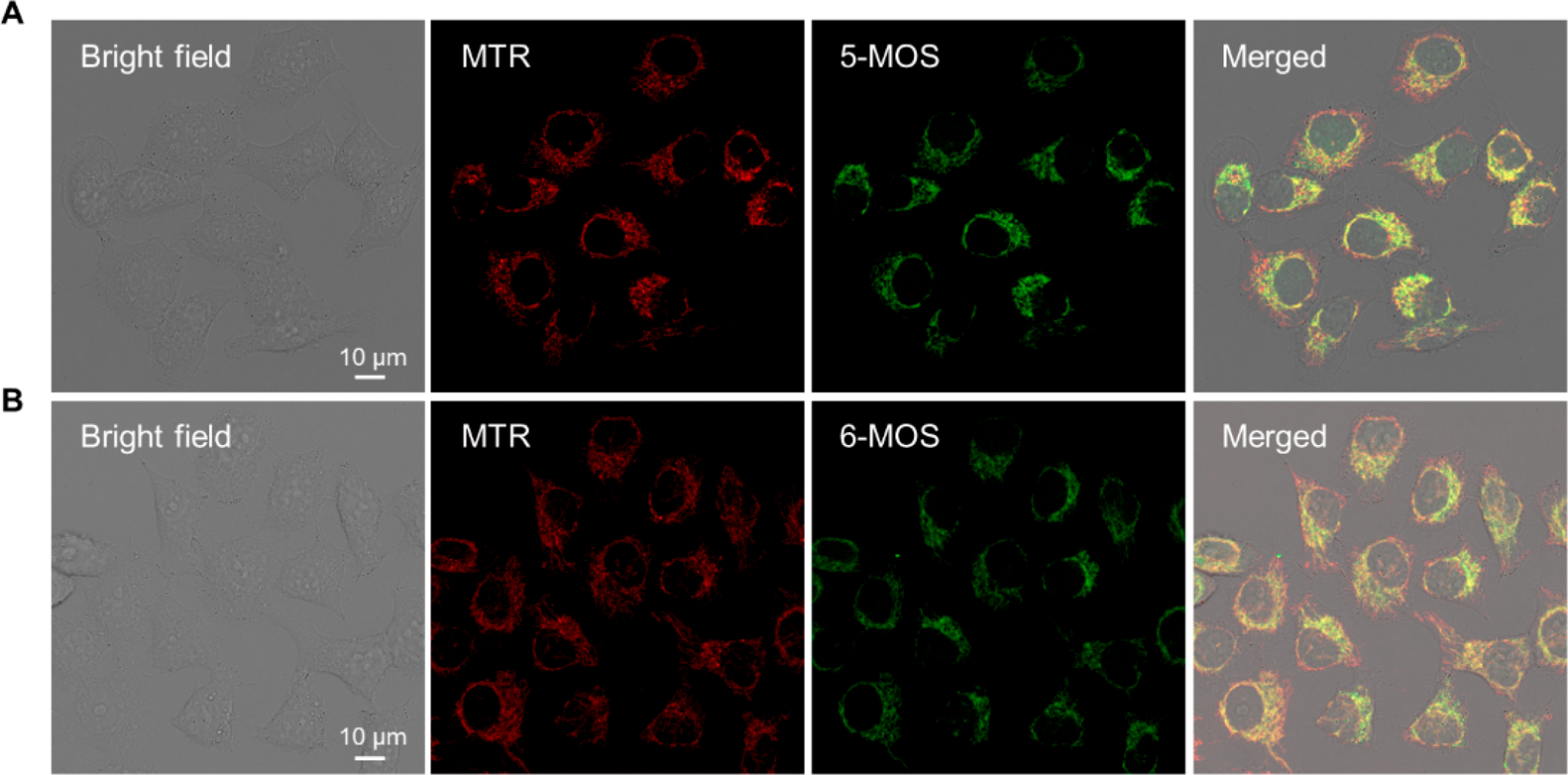
Colocalization imaging analysis of 5-MOS and 6-MOS. (A) Confocal images of MHCC97H cells stained with MTR for 15 min and 5-MOS for 15 min. (B) Confocal images of MHCC97H cells stained with MTR for 15 min and 6-MOS for 1.5 h. Concentration of MTR: 50 nM. Concentration of 5-MOS and 6-MOS: 10 μM. Excitation wavelength: 405 nm for 5-MOS and 6-MOS and 561 nm for MTR.

The solid-state luminescence of 5-MOS and 6-MOS and their innate excellent bioactivities encouraged us to explore their application in cell imaging. First, the cytotoxicity of them was evaluated using a Cell-Counting-Kit-8 (CCK-8) assay, and no significant variation in the cell viability was observed even at the concentration of 20 μM (Figure S19), suggesting low cytotoxicity and good biocompatibility of the two natural coumarins. Then, the live cell imaging experiments were carried out. As shown in Figure 5, the MHCC97H cells stained with 5-MOS could be observed clearly with negligible background fluorescence, while the cells stained with 6-MOS, in sharp contrast, could hardly be seen without washing procedures due to the interference of strong background fluorescence. The results indicated that 5-MOS was a wash-free probe, while 6-MOS was a typical “always-on” probe, requiring tedious and time-consuming washing procedures. It is worthy to note that traditional molecular rotor based AIEgens could also realize wash-free imaging when the nonemissive molecular species aggregated within cells to light up the emission.^64^ However, the wash-free imaging of 5-MOS does not follow a similar mechanism, since the molecular species of 5-MOS are brightly emissive, while the aggregates in aqueous media are nonemissive. Based on the fluorescence property of 5-MOS, we think that the nonemissive aggregates in aqueous media may disaggregate within cells and interact with the biomacromolecules in cells to light up the emission. To confirm our conjecture, bovine serum albumin (BSA) was chosen to simulate the fluorophore-protein interactions. As shown in Figure S20, with increasing the concentration of BSA proteins in 5-MOS solution, the fluorescence of 5-MOS increased gradually with a blueshift of the maximum emission wavelength from 518 to 466 nm, which matched with the emission wavelength of molecular species of 5-MOS (473 nm). These results support our conjecture that the aggregates of 5-MOS may disaggregate in the cells and interact with the cellular proteins to light up the emission. Therefore, 5-MOS demonstrates a new strategy to achieve wash-free imaging. To further determine the target site of the two coumarins in cells, colocalization imaging experiments were conducted in MHCC97H cells with Mito Tracker Red (MTR), a commercial mitochondria-specific probe, used as a reference. As shown in Figure 6, the green fluorescence from 5-MOS and 6-MOS was overlapped with the red fluorescence obtained by MTR with Pearson correlation coefficients of 0.7345 and 0.7447, respectively, indicating that the two coumarins mainly target mitochondria. This could be further confirmed by similar results obtained in HEL-1 cells (Figure S21).

## Conclusion

In summary, 5-MOS and 6-MOS, a pair of AIE coumarin isomers, were discovered from the fluorescent roots of medicinal plant *T. asiatica*. In spite of the slight difference in molecular structures, the two isomers exhibit completely contrary fluorescent properties upon aggregation in aqueous media. Crystal analyses as well as theoretical calculations indicate that the AIE properties of 6-MOS could be elegantly explained by the mechanism of restriction of intramolecular motion (RIM). Also, the unusual quenching effect of 5-MOS in an aqueous environment was because of the electron/energy transfer among 5-MOS molecules and the different aggregates. In crystals, without the inference of water molecules, 5-MOS molecules packed closely with each other to form a uniform assembly, thus affording the bright emission. Interestingly, the quenched fluorescence of 5-MOS in a water system can be switched on when binding with proteins, which can enable its application as a wash-free probe of cellular mitochondria. In general, this work not only presents an ingenious tactic to seek novel AIEgens efficiently from natural fluorescent species but also provides an idea to study AIE-active molecules with strong emission in crystals but weak emission in the aggregate state.

## Supporting Information Available

Materials, experimental and computational details, and related supporting data, including Figures S1–S21 and Tables S1–S3 (PDF)
Video S1: Drastic intramolecular vibrations of 6-MOS in excited state by DFT calculation (MP4)

## References

[ref1] Kim B. H.; Li K.; Kim J.-T.; Park Y.; Jang H.; Wang X.; Xie Z.; Won S. M.; Yoon H.-J.; Lee G.; Jang W. J.; Lee K. H.; Chung T. S.; Jung Y. H.; Heo S. Y.; Lee Y.; Kim J.; Cai T.; Kim Y.; Prasopsukh P.; Yu Y.; Yu X.; Avila R.; Luan H.; Song H.; Zhu F.; Zhao Y.; Chen L.; Han S. H.; Kim J.; Oh S. J.; Lee H.; Lee C. H.; Huang Y.; Chamorro L. P.; Zhang Y.; Rogers J. A. Three-dimensional electronic microfliers inspired by wind-dispersed seeds. Nature 2021, 597 (7877), 503–524. 10.1038/s41586-021-03847-y. 34552257

[ref2] Schaffner M.; Faber J. A.; Pianegonda L.; Ruhs P. A.; Coulter F.; Studart A. R. 3D printing of robotic soft actuators with programmable bioinspired architectures. Nat. Commun. 2018, 9, 878 10.1038/s41467-018-03216-w. 29491371 PMC5830454

[ref3] Lu H.; Zhang M.; Yang Y.; Huang Q.; Fukuda T.; Wang Z.; Shen Y. A bioinspired multilegged soft millirobot that functions in both dry and wet conditions. Nat. Commun. 2018, 9, 3944 10.1038/s41467-018-06491-9. 30258072 PMC6158235

[ref4] Shi X.; Dou R.; Ma T.; Liu W.; Lu X.; Shea K. J.; Song Y.; Jiang L. Bioinspired Lotus-like Self-Illuminous Coating. ACS Appl. Mater. Interfaces 2015, 7 (33), 18424–18428. 10.1021/acsami.5b04499. 26238797

[ref5] Zhou H.; Xu J.; Liu X.; Zhang H.; Wang D.; Chen Z.; Zhang D.; Fan T. Bio-Inspired Photonic Materials: Prototypes and Structural Effect Designs for Applications in Solar Energy Manipulation. Adv. Funct. Mater. 2018, 28 (24), 1705309 10.1002/adfm.201705309.

[ref6] Weiss P. S. 2008 Nobel Prize in Chemistry: Green Fluorescent Protein, Its Variants and Implications. ACS Nano 2008, 2 (10), 1977 10.1021/nn800671h. 19206439

[ref7] Heim R.; Prasher D. C.; Tsien R. Y. Wavelength mutations and posttranslational autoxidation of green fluorescent protein. Proc. Natl. Acad. Sci. U. S. A. 1994, 91 (26), 12501–12504. 10.1073/pnas.91.26.12501. 7809066 PMC45466

[ref8] Jiang M.; Gu X.; Lam J. W. Y.; Zhang Y.; Kwok R. T. K.; Wong K. S.; Tang B. Z. Two-photon AIE bio-probe with large Stokes shift for specific imaging of lipid droplets. Chem. Sci. 2017, 8 (8), 5440–5446. 10.1039/C7SC01400G. 28970923 PMC5609514

[ref9] Jiang M.; He Z.; Zhang Y.; Sung H. H. Y.; Lam J. W. Y.; Peng Q.; Yan Y.; Wong K. S.; Williams I. D.; Zhao Y.; Tang B. Z. Development of benzylidene-methyloxazolone based AIEgens and decipherment of their working mechanism. J. Mater. Chem. C 2017, 5 (29), 7191–7199. 10.1039/C7TC02582C.

[ref10] Walker C. L.; Lukyanov K. A.; Yampolsky I. V.; Mishin A. S.; Bommarius A. S.; Duraj-Thatte A. M.; Azizi B.; Tolbert L. M.; Solntsev K. M. Fluorescence imaging using synthetic GFP chromophores. Curr. Opin. Chem. Biol. 2015, 27, 64–74. 10.1016/j.cbpa.2015.06.002. 26117808

[ref11] Duval R.; Duplais C. Fluorescent natural products as probes and tracers in biology. Nat. Prod. Rep. 2017, 34 (2), 161–193. 10.1039/C6NP00111D. 28125109

[ref12] Li Q.; Gong J.; Li Y.; Zhang R.; Wang H.; Zhang J.; Yan H.; Lam J. W. Y.; Sung H. H. Y.; Williams I. D.; Kwok R. T. K.; Li M.-H.; Wang J.; Tang B. Z. Unusual light-driven amplification through unexpected regioselective photogeneration of five-membered azaheterocyclic AIEgen. Chem. Sci. 2021, 12 (2), 709–717. 10.1039/D0SC04725B. PMC8179000 34163804

[ref13] Wang H.; Li Q.; Zhang J.; Zhang H.; Shu Y.; Zhao Z.; Jiang W.; Du L.; Phillips D. L.; Lam J. W. Y.; Sung H. H. Y.; Williams I. D.; Lu R.; Tang B. Z. Visualization and Manipulation of Solid-State Molecular Motions in Cocrystallization Processes. J. Am. Chem. Soc. 2021, 143 (25), 9468–9477. 10.1021/jacs.1c02594. 34152134

[ref14] Kato S.; Furukawa S.; Aoki D.; Goseki R.; Oikawa K.; Tsuchiya K.; Shimada N.; Maruyama A.; Numata K.; Otsuka H. Crystallization-induced mechanofluorescence for visualization of polymer crystallization. Nat. Commun. 2021, 12 (1), 126 10.1038/s41467-020-20366-y. 33402691 PMC7785725

[ref15] Luo J. D.; Xie Z. L.; Lam J. W. Y.; Cheng L.; Chen H. Y.; Qiu C. F.; Kwok H. S.; Zhan X. W.; Liu Y. Q.; Zhu D. B.; Tang B. Z. Aggregation-induced emission of 1-methyl-1,2,3,4,5-pentaphenylsilole. Chem. Commun. 2001, (18), 1740–1741. 10.1039/b105159h. 12240292

[ref16] Wuerthner F. Aggregation-Induced Emission (AIE): A Historical Perspective. Angew. Chem., Int. Ed. 2020, 59 (34), 14192–14196. 10.1002/anie.202007525. 32662175

[ref17] Schmidt G. Über Lumineszenz von festen Lösungen. Ann. Phys. Berlin. 1921, 370 (11), 247–256. 10.1002/andp.19213701105.

[ref18] Stark J.; Lipp P. The effect of inner molecular relative movement on the intensity of absorption of fluorescent by valence electrons. Z. Phys. Chem. 1913, 86, 36–41.

[ref19] Schmidt G. BeiWilye sur Eenntniss der Plzcorescens. Ann. Phys. Berlin. 1896, 294, 103–130. 10.1002/andp.18962940507.

[ref20] Chen Y. C.; Lam J. W. Y.; Kwok R. T. K.; Liu B.; Tang B. Z. Aggregation-induced emission: fundamental understanding and future developments. Mater. Horiz. 2019, 6 (3), 428–433. 10.1039/C8MH01331D.

[ref21] Gu X. G.; Zhang D. Q. Reaction: The Impact of the AIE Concept. Chem. 2020, 6 (6), 1199–1200. 10.1016/j.chempr.2020.05.019.

[ref22] Tu Y. J.; Zhao Z.; Lam J. W. Y.; Tang B. Z. Mechanistic connotations of restriction of intramolecular motions (RIM). Natl. Sci. Rev. 2021, 8 (6), nwaa260 10.1093/nsr/nwaa260. 34691663 PMC8288185

[ref23] Guan J. X.; Shen C. Z.; Peng J.; Zheng J. R. What Leads to Aggregation-Induced Emission?. J. Phys. Chem. Lett. 2021, 12 (17), 4218–4226. 10.1021/acs.jpclett.0c03861. 33900751

[ref24] Guan J. X.; Wei R.; Prlj A.; Peng J.; Lin K. H.; Liu J. T.; Han H.; Corminboeuf C.; Zhao D. H.; Yu Z. H.; Zheng J. R. Direct Observation of Aggregation-Induced Emission Mechanism. Angew. Chem., Int. Ed. 2020, 59 (35), 14903–14909. 10.1002/anie.202004318. 32441469

[ref25] Prlj A.; Doslic N.; Corminboeuf C. How does tetraphenylethylene relax from its excited states?. Phys. Chem. Chem. Phys. 2016, 18 (17), 11606–11609. 10.1039/C5CP04546K. 26395765

[ref26] Li Q. S.; Blancafort L. A conical intersection model to explain aggregation induced emission in diphenyl dibenzofulvene. Chem. Commun. 2013, 49 (53), 5966–5968. 10.1039/c3cc41730a. 23715286

[ref27] Zhang J.; Hu L.; Zhang K.; Liu J.; Li X.; Wang H.; Wang Z.; Sung H. H. Y.; Williams I. D.; Zeng Z.; Lam J. W. Y.; Zhang H.; Tang B. Z. How to Manipulate Through-Space Conjugation and Clusteroluminescence of Simple AIEgens with Isolated Phenyl Rings. J. Am. Chem. Soc. 2021, 143 (25), 9565–9574. 10.1021/jacs.1c03882. 34115474

[ref28] Gong J. Y.; Gong W. J.; Wu B.; Wang H. R.; He W.; Dai Z. Y.; Li Y. Z.; Liu Y.; Wang Z. M.; Tuo X. J.; Lam J. W. Y.; Qiu Z. J.; Zhao Z.; Tang B. Z. ASBase: The universal database for aggregate science. Aggregate 2023, 4, e263 10.1002/agt2.263.

[ref29] Sasaki S.; Suzuki S.; Sameera W. M. C.; Igawa K.; Morokuma K.; Konishi G. Highly Twisted N,N-Dialkylamines as a Design Strategy to Tune Simple Aromatic Hydrocarbons as Steric Environment-Sensitive Fluorophores. J. Am. Chem. Soc. 2016, 138 (26), 8194–8206. 10.1021/jacs.6b03749. 27300152

[ref30] Sturala J.; Etherington M. K.; Bismillah A. N.; Higginbotham H. F.; Trewby W.; Aguilar J. A.; Bromley E. H. C.; Avestro A. J.; Monkman A. P.; McGonigal P. R. Excited-State Aromatic Interactions in the Aggregation-Induced Emission of Molecular Rotors. J. Am. Chem. Soc. 2017, 139 (49), 17882–17889. 10.1021/jacs.7b08570. 29151342

[ref31] Liu J.; Zhang H.; Hu L.; Wang J.; Lam J. W. Y.; Blancafort L.; Tang B. Z. Through-Space Interaction of Tetraphenylethylene: What, Where, and How. J. Am. Chem. Soc. 2022, 144 (17), 7901–7910. 10.1021/jacs.2c02381. 35443776

[ref32] Suzuki S.; Sasaki S.; Sairi A. S.; Iwai R.; Tang B. Z.; Konishi G.-i. Principles of Aggregation-Induced Emission: Design of Deactivation Pathways for Advanced AIEgens and Applications. Angew. Chem., Int. Ed. 2020, 59 (25), 9856–9867. 10.1002/anie.202000940. PMC7318703 32154630

[ref33] Xu S.; Duan Y.; Liu B. Precise Molecular Design for High-Performance Luminogens with Aggregation-Induced Emission. Adv. Mater. 2020, 32 (1), 1903530 10.1002/adma.201903530. 31583787

[ref34] Zhao W.; He Z.; Peng Q.; Lam J. W. Y.; Ma H.; Qiu Z.; Chen Y.; Zhao Z.; Shuai Z.; Dong Y.; Tang B. Z. Highly sensitive switching of solid-state luminescence by controlling intersystem crossing. Nat. Commun. 2018, 9, 3044 10.1038/s41467-018-05476-y. 30072690 PMC6072740

[ref35] Micheletti C.; Wang Q.; Ventura F.; Turelli M.; Ciofini I.; Adamo C.; Pucci A. Red-emitting tetraphenylethylene derivative with aggregation-induced enhanced emission for luminescent solar concentrators: A combined experimental and density functional theory study. Aggregate 2022, 3 (2), e188 10.1002/agt2.188.

[ref36] Zhong D.; Chen W.; Xia Z.; Hu R.; Qi Y.; Zhou B.; Li W.; He J.; Wang Z.; Zhao Z.; Ding D.; Tian M.; Tang B. Z.; Zhou M. Aggregation-induced emission luminogens for image-guided surgery in non-human primates. Nat. Commun. 2021, 12 (1), 6485 10.1038/s41467-021-26417-2. 34759280 PMC9632329

[ref37] Li Y.; Cai Z.; Liu S.; Zhang H.; Wong S. T. H.; Lam J. W. Y.; Kwok R. T. K.; Qian J.; Tang B. Z. Design of AIEgens for near-infrared IIb imaging through structural modulation at molecular and morphological levels. Nat. Commun. 2020, 11 (1), 1255 10.1038/s41467-020-15095-1. 32152288 PMC7062876

[ref38] Liu M.; Chen Y.; Guo Y.; Yuan H.; Cui T.; Yao S.; Jin S.; Fan H.; Wang C.; Xie R.; He W.; Guo Z. Golgi apparatus-targeted aggregation-induced emission luminogens for effective cancer photodynamic therapy. Nat. Commun. 2022, 13 (1), 2179 10.1038/s41467-022-29872-7. 35449133 PMC9023483

[ref39] Cai X.; Liu B. Aggregation-Induced Emission: Recent Advances in Materials and Biomedical Applications. Angew. Chem., Int. Ed. 2020, 59 (25), 9868–9886. 10.1002/anie.202000845. 32128951

[ref40] Qian J.; Tang B. Z. AIE Luminogens for Bioimaging and Theranostics: from Organelles to Animals. Chem. 2017, 3 (1), 56–91. 10.1016/j.chempr.2017.05.010.

[ref41] Kenry; Tang B. Z.; Liu B. Catalyst: Aggregation-Induced Emission-How Far Have We Come, and Where Are We Going Next?. Chem. 2020, 6 (6), 1195–1198. 10.1016/j.chempr.2020.05.018.

[ref42] Gu Y.; Zhao Z.; Su H.; Zhang P.; Liu J.; Niu G.; Li S.; Wang Z.; Kwok R. T. K.; Ni X.-L.; Sun J.; Qin A.; Lam J. W. Y.; Tang B. Z. Exploration of biocompatible AIEgens from natural resources. Chem. Sci. 2018, 9 (31), 6497–6502. 10.1039/C8SC01635F. 30310579 PMC6115644

[ref43] He T.; Niu N.; Chen Z.; Li S.; Liu S.; Li J. Novel Quercetin Aggregation-Induced Emission Luminogen (AIEgen) with Excited-State Intramolecular Proton Transfer for In Vivo Bioimaging. Adv. Funct. Mater. 2018, 28 (11), 1706196 10.1002/adfm.201706196.

[ref44] Cai X.-M.; Lin Y.; Li Y.; Chen X.; Wang Z.; Zhao X.; Huang S.; Zhao Z.; Tang B. Z. BioAIEgens derived from rosin: how does molecular motion affect their photophysical processes in solid state?. Nat. Commun. 2021, 12 (1), 1773 10.1038/s41467-021-22061-y. 33741995 PMC7979920

[ref45] Cao D.; Liu Z.; Verwilst P.; Koo S.; Jangjili P.; Kim J. S.; Lin W. Coumarin-Based Small-Molecule Fluorescent Chemosensors. Chem. Rev. 2019, 119 (18), 10403–10519. 10.1021/acs.chemrev.9b00145. 31314507

[ref46] Bu F.; Duan R.; Xie Y.; Yi Y.; Peng Q.; Hu R.; Qin A.; Zhao Z.; Tang B. Z. Unusual Aggregation-Induced Emission of a Coumarin Derivative as a Result of the Restriction of an Intramolecular Twisting Motion. Angew. Chem., Int. Ed. 2015, 54 (48), 14492–14497. 10.1002/anie.201506782. 26439884

[ref47] Luo Z.; Liu T.; Yan H.; Zou Y.; Yang C. Isomerization Strategy of Nonfullerene Small-Molecule Acceptors for Organic Solar Cells. Adv. Funct. Mater. 2020, 30 (46), 2004477 10.1002/adfm.202004477.

[ref48] Inoue S.; Higashino T.; Arai S.; Kumai R.; Matsui H.; Tsuzuki S.; Horiuchi S.; Hasegawa T. Regioisomeric control of layered crystallinity in solution-processable organic semiconductors. Chem. Sci. 2020, 11 (46), 12493–12505. 10.1039/D0SC04461J. 34976335 PMC8647348

[ref49] Shao B.; Qian H.; Li Q.; Aprahamian I. Structure Property Analysis of the Solution and Solid-State Properties of Bistable Photochromic Hydrazones. J. Am. Chem. Soc. 2019, 141 (20), 8364–8371. 10.1021/jacs.9b03932. 31050408

[ref50] Jiang H.; Hu P.; Ye J.; Chaturvedi A.; Zhang K. K.; Li Y.; Long Y.; Fichou D.; Kloc C.; Hu W. From Linear to Angular Isomers: Achieving Tunable Charge Transport in Single-Crystal Indolocarbazoles Through Delicate Synergetic CH/NH Interactions. Angew. Chem., Int. Ed. 2018, 57 (29), 8875–8880. 10.1002/anie.201713288. 29457325

[ref51] Kurata R.; Ito A.; Gon M.; Tanaka K.; Chujo Y. Diarylamino- and Diarylboryl-Substituted Donor-Acceptor Pyrene Derivatives: Influence of Substitution Pattern on Their Photophysical Properties. J. Org. Chem. 2017, 82 (10), 5111–5121. 10.1021/acs.joc.7b00315. 28481543

[ref52] Xiong Y.; Zhao Z.; Zhao W.; Ma H.; Peng Q.; He Z.; Zhang X.; Chen Y.; He X.; Lam J. W. Y.; Tang B. Z. Designing Efficient and Ultralong Pure Organic Room-Temperature Phosphorescent Materials by Structural Isomerism. Angew. Chem., Int. Ed. 2018, 57 (27), 7997–8001. 10.1002/anie.201800834. 29736955

[ref53] Zeng Z.; Tian R.; Feng J.; Yang N.; Yuan L. A systematic review on traditional medicine *Toddalia asiatica* (L.) Lam.: Chemistry and medicinal potential. Saudi Pharm. J. 2021, 29 (8), 781–798. 10.1016/j.jsps.2021.05.003. 34408540 PMC8360773

[ref54] Tang Z.-H.; Liu Y.-B.; Ma S.-G.; Li L.; Li Y.; Jiang J.-D.; Qu J.; Yu S.-S. Antiviral Spirotriscoumarins A and B: Two Pairs of Oligomeric Coumarin Enantiomers with a Spirodienone-Sesquiterpene Skeleton from *Toddalia asiatica*. Org. Lett. 2016, 18 (19), 5146–5149. 10.1021/acs.orglett.6b02572. 27673343

[ref55] Ryu N.; Okazaki Y.; Pouget E.; Takafuji M.; Nagaoka S.; Ihara H.; Oda R. Fluorescence emission originated from the H-aggregated cyanine dye with chiral gemini surfactant assemblies having a narrow absorption band and a remarkably large Stokes shift. Chem. Commun. 2017, 53 (63), 8870–8873. 10.1039/C7CC04484D. 28737786

[ref56] Lai T. I.; Lim B. T.; Lim E. C. Photophysical Properties of Biological Important Molecules Related to Proximity Effects: Psoralens. J. Am. Chem. Soc. 1982, 104 (26), 7631–7635. 10.1021/ja00390a039.

[ref57] Tu Y.; Yu Y.; Xiao D.; Liu J.; Zhao Z.; Liu Z.; Lam J. W. Y.; Tang B. Z. An Intelligent AIEgen with Nonmonotonic Multiresponses to Multistimuli. Adv. Sci. 2020, 7 (20), 2001845 10.1002/advs.202001845. PMC7578909 33101873

[ref58] Zhao G.-J.; Han K.-L. Hydrogen Bonding in the Electronic Excited State. Acc. Chem. Res. 2012, 45 (3), 404–413. 10.1021/ar200135h. 22070387

[ref59] Huang G.-J.; Ho J.-H.; Prabhakar C.; Liu Y.-H.; Peng S.-M.; Yang J.-S. Site-Selective Hydrogen-Bonding-Induced Fluorescence Quenching of Highly Solvatofluorochromic GFP-like Chromophores. Org. Lett. 2012, 14 (19), 5034–5037. 10.1021/ol302237k. 22985356

[ref60] Xie S.; Manuguri S.; Ramstrom O.; Yan M. Impact of Hydrogen Bonding on the Fluorescence of N-Amidinated Fluoroquinolones. Chem. Asian J. 2019, 14 (6), 910–916. 10.1002/asia.201801916. 30762939

[ref61] Wu M.; Mukherjee P.; Lamont D. N.; Waldeck D. H. Electron Transfer and Fluorescence Quenching of Nanoparticle Assemblies. J. Phys. Chem. C 2010, 114 (13), 5751–5759. 10.1021/jp9098667.

[ref62] Koole R.; Liljeroth P.; de Mello Donegá C.; Vanmaekelbergh D.; Meijerink A. Electronic coupling and exciton energy transfer in CdTe quantum-dot molecules. J. Am. Chem. Soc. 2006, 128 (32), 10436–10441. 10.1021/ja061608w. 16895408

[ref63] Fan Q.; Li L.; Xue H.; Zhou H.; Zhao L.; Liu J.; Mao J.; Wu S.; Zhang S.; Wu C.; Li X.; Zhou X.; Wang J. Precise Control Over Kinetics of Molecular Assembly: Production of Particles with Tunable Sizes and Crystalline Forms. Angew. Chem., Int. Ed. 2020, 59 (35), 15141–15146. 10.1002/anie.202003922. 32432368

[ref64] Chen Y.; Zhang W.; Zhao Z.; Cai Y.; Gong J.; Kwok R. T. K.; Lam J. W. Y.; Sung H. H. Y.; Williams I. D.; Tang B. Z. An Easily Accessible Ionic Aggregation-Induced Emission Luminogen with Hydrogen-Bonding-Switchable Emission and Wash-Free Imaging Ability. Angew. Chem., Int. Ed. 2018, 57 (18), 5011–5015. 10.1002/anie.201800772. 29512250

