# Supplementary material for: Natural Coumarin Isomers with Dramatically Different AIE Properties: Mechanism and Application
Source: ACS Cent Sci. 2023 Apr 19;9(5):883–91. doi: 10.1021/acscentsci.3c00012 (PMC10214507; doi:10.1021/acscentsci.3c00012)
Supplement: Supplementary file 1 — oc3c00012_si_001.pdf [file oc3c00012_si_001.pdf]

# Supporting Information

## Natural Coumarin Isomers with Dramatically Different AIE property: Mechanism and Application

Shan-Shan Chen,[1,3,5,†], Haoran Wang,[2,4,†] Bo Wu,[2] Qiyao Li,[2] Junyi Gong,[2] Yun-Li Zhao,[3] Yun Zhao,[1,5] Xia Xiao,[3] Jacky W. Y. Lam,[4] Zheng Zhao,[2]* Xiao-Dong Luo,[1,3]* and Ben Zhong Tang[2,4,]*

[1]State Key Laboratory of Phytochemistry and Plant Resources in West China, Kunming Institute of Botany, Chinese Academy of Sciences, Kunming 650201, PR China.

[2]School of Science and Engineering, Shenzhen Institute of Aggregate Science and Technology, The Chinese University of Hong Kong, Shenzhen, Guangdong 518172, China.

[3]Key Laboratory of Medicinal Chemistry for Natural Resource, Ministry of Education and Yunnan Province, Yunnan Characteristic Plant Extraction Laboratory, School of Chemical Science and Technology, Yunnan University, Kunming, 650500, PR China

[4]Hong Kong Branch of Chinese National Engineering Research Center for Tissue Restoration and Reconstruction and Department of Chemistry, The Hong Kong University of Science and Technology, Clear Water Bay, Kowloon, Hong Kong, China.

[5]University of Chinese Academy of Sciences, Beijing 100049, PR China.

*Corresponding authors. E-mails: zhaozheng@cuhk.edu.cn (Z.Z.), xdluo@mail.kib.ac.cn (X.-D.L.), tangbenz@cuhk.edu.cn (B.Z.T.)

[†]Equally contributed to this work

# Table of Contents

Experimental Procedures ....................3

General information ....................3
Preparation procedures and characterization data ....................3
Theoretical calculation methods ....................4
Cell culture ....................5
Cell imaging ....................5
Cell viability Assay ....................6

Supplementary Figures and Tables ....................6

Figure S1. ....................7
Figure S2. ....................7
Figure S3. ....................7
Table S1. ....................9
Figure S4. ....................10
Figure S5. ....................10
Figure S6. ....................11
Table S2. ....................11
Table S3. ....................12
Figure S7. ....................12
Figure S8. ....................13
Figure S9. ....................13
Figure S10. ....................13
Figure S11. ....................14
Figure S12. ....................15
Figure S13. ....................16
Figure S15. ....................17
Figure S16. ....................17
Figure S17. ....................18
Figure S18. ....................18
Figure S19. ....................19
Figure S20. ....................19
Figure S21. ....................20

Reference ....................20

# Experimental Procedures

## General information

The dried roots of *Toddalia asiatica* were purchased from Luo-Si-Wan herbal medicine market of Kunming, Yunnan province, China in May 2020 (origin: Hunan province) and identified by Mr. Jun Zhang (Kunming Plant Classification Biotechnology Co., Ltd.). A voucher specimen (No. 2020051801) was deposited at the School of Chemical Science and Technology, Yunnan University, China. $^1H$ and $^{13}C$ NMR spectra were recorded on a Bruker AVANCE III 400 MHz spectrometer with TMS as the internal standard. HRESIMS data were measured on an Agilent 1290 UPLC/6540 Q-TOF spectrometer. UV/vis absorption spectra were measured on Shimadzu UV-2700 spectrophotometer. The photoluminescence (PL) spectra were recorded on a F-7000 fluorescence spectrophotometer. The absolute fluorescence quantum yield (PLQY) was measured by absolute PL quantum yield measurement system C9920-02. The PL decay curves were measured using Quantaurus-Tau. Single-crystal data was collected on a Bruker D8 VENTURE X-ray single crystal diffractometer with Cu Kα radiation. The X-ray diffraction pattern was collected on a Rigaku RINT-TTR III X-ray diffractometer with Cu Kα radiation. Dynamic light scattering (DLS) data were measured by Malvern Zetasizer Nano ZS instruments. No unexpected or unusually high safety hazards were encountered.

## Preparation procedures and characterization data

Air-dried roots of *Toddalia asiatica* (10.0 kg) were extracted with 90% methanol under reflux to obtain a crude extract (1.8 kg). The crude extract was suspended in 0.5% aqueous hydrochloric acid (v/v) and then partitioned with ethyl acetate. Guided by the blue fluorescence, the ethyl acetate fraction (1.0 kg) was chromatographed on a silica gel column eluted with a gradient of petroleum ether-ethyl acetate (10:1→0:10, v/v) to

yield 5-methoxyseselin (49.2 mg) and braylin (6-methoxyseselin) (236.0 mg). The crystal of 5-methoxyseselin for X-ray crystallographic measurements were obtained by slow evaporation from a chloroform/methanol mixture, and the crystal of braylin for X-ray crystallographic measurements were obtained by slow evaporation from a petroleum ether/ethyl acetate mixture.

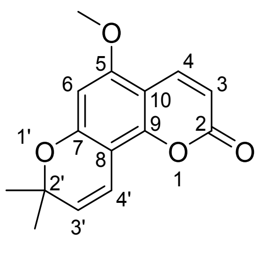


**5-Methoxyseselin：** white amorphous powder; $C_{15}H_{14}O_4$; $^{1}H$ NMR (400 MHz, $CDCl_3$): $\delta$ 7.93 (1H, d, $J$ = 9.6 Hz, H-4), 6.77 (1H, d, $J$ = 10.0 Hz, H-4′), 6.22 (1H, s, H-6), 6.11 (1H, d, $J$ = 9.6 Hz, H-3), 5.57 (1H, d, $J$ = 10.0 Hz, H-3′), 3.87 (3H, s, $OCH_3$-5), 1.46 (6H, s, 2×$CH_3$); $^{13}C$ NMR (100 MHz, $CDCl_3$): $\delta$ 161.3 (s, C-2), 157.4 (s, C-7), 156.5 (s, C-5), 151.0 (s, C-9), 139.0 (d, C-4), 127.5 (d, C-3′), 114.9 (d, C-4′), 110.3 (d, C-3), 103.6 (s, C-10), 102.5 (s, C-8), 95.4 (d, C-6), 78.0 (s, C-2′), 56.0 (q, $OCH_3$-5), 28.1 (q, 2×$CH_3$). HRMS (ESI): m/z 259.0966 $[M + H]^+$ (calcd for $C_{15}H_{15}O_4^+$, 259.0965).

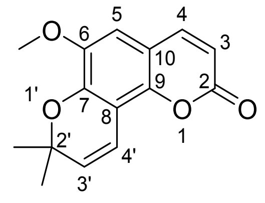


**Braylin (6-methoxyseselin)：** white amorphous powder; $C_{15}H_{14}O_4$; $^{1}H$ NMR (400 MHz, $CDCl_3$): $\delta$ 7.63 (1H, d, $J$ = 9.5 Hz, H-4), 6.83 (1H, s, H-5), 6.82 (1H, d, $J$ = 9.8 Hz, H-4′), 6.23 (1H, d, $J$ = 9.5 Hz, H-3), 5.76 (1H, d, $J$ = 10.0 Hz, H-3′), 3.89 (3H, s, $OCH_3$-6), 1.52 (6H, s, 2×$CH_3$); $^{13}C$ NMR (100 MHz, $CDCl_3$): $\delta$ 160.9 (s, C-2), 145.7 (s, C-6), 145.5 (s, C-7), 144.6 (s, C-9), 143.8 (d, C-4), 130.7 (d, C-3′), 114.9 (d, C-4′), 112.8 (d, C-3), 111.3 (s, C-10), 109.9 (s, C-8), 108.7 (d, C-5), 77.8 (s, C-2′), 56.3 (q, $OCH_3$-6), 27.8 (q, 2×$CH_3$). HRMS (ESI): m/z 259.0965 $[M + H]^+$ (calcd for $C_{15}H_{15}O_4^+$, 259.0965).

**Theoretical calculation methods**

The equilibrim conformations of 5-MOS and 6-MOS molecules were optimized

through DFT calculation on BLYP/def2-SVP level with Grimme's D4 correction and the solvent model of density (SMD). The natural bond orbital analyses of them were performed on NBO 6.0 with wavefunction information provided by DFT calculation. These DFT calculations were carried out with ORCA (ver. 5.0.1) quantum mechianics suite. The independent gradient model based on Hirshfeld partition (IGMH) method in Multiwfn 3.8 program was employed to study the weak intermolecular interactions between 5-MOS and water molecules [1]. The ground and excited state geometry optimizations of 5-MOS-3$H_2O$ complex were carried out at the B3LYP-D3/def2-TZVP(-f) and B3LYP-D3/def2-SVP level, respectively. These calculations were performed using the ORCA 5.0 quantum chemistry program package from the development team at the Max Planck Institute for Bioinorganic Chemistry [2].

**Cell culture**

MHCC97H and HEL-1 cell lines were obtained from ATCC and cultured in dulbecco's modified eagle medium (DMEM) media with 10% fetal bovine serum (FBS), 100 U/ml penicillin/streptomycin in a humidified incubator containing 5% $CO_2$ at 37°C. The cells were sub-cultured when they nearly reached confluence.

**Cell imaging**

For cell imaging, the cells were seeded on 35 mm confocal dishes and grown until adherent well at 37 ℃ with 5% $CO_2$. The cells were incubated with 5-MOS/6-MOS at the concentration of 10 μM (by adding 10 μL of 2 mM stock solution in DMSO to a 2 mL of DMEM media with DMSO = 0.5 vol %) for 15 min/1.5h. To investigate their distribution in living cells, commercial mitochondrial dyes Mito Tracker Red (MTR) was used at the concentration of 50 nM for 15 min. After washed by DMEM for three times, the cells were imaged under a confocal microscope (Leica TCS SP8 laser scanning confocal microscope) using proper excitation and emission filters for each dye:

for 5-MOS and 6-MOS, the excitation filter was 405 nm and the emission filter 410−550 nm; for MTR, the excitation filter was 561 nm and the emission filter 566−650 nm.

### Cell viability Assay

The cell viability treated by 5-MOS and 6-MOS was determined using the Cell Counting Kit-8 (CCK-8) assay. MHCC97H and HEL-1 cells were seeded into 96-well plates at a density of 5 × $10^3$ cells per well (100 μL/well) and grown for 24 h. Then another 100 μL new DMEM medium containing different concentrations of 5-MOS and 6-MOS were add into each well. After 24 h incubation, the culture medium was discarded and 100 μL new DMEM medium containing 10 μL CCK-8 solution was added to the wells and incubated for another 1 h. The absorbance at 450 nm was recorded by SPECTRAMAX 190 microplate reader (Gemini XPS).

## Supplementary Figures and Tables

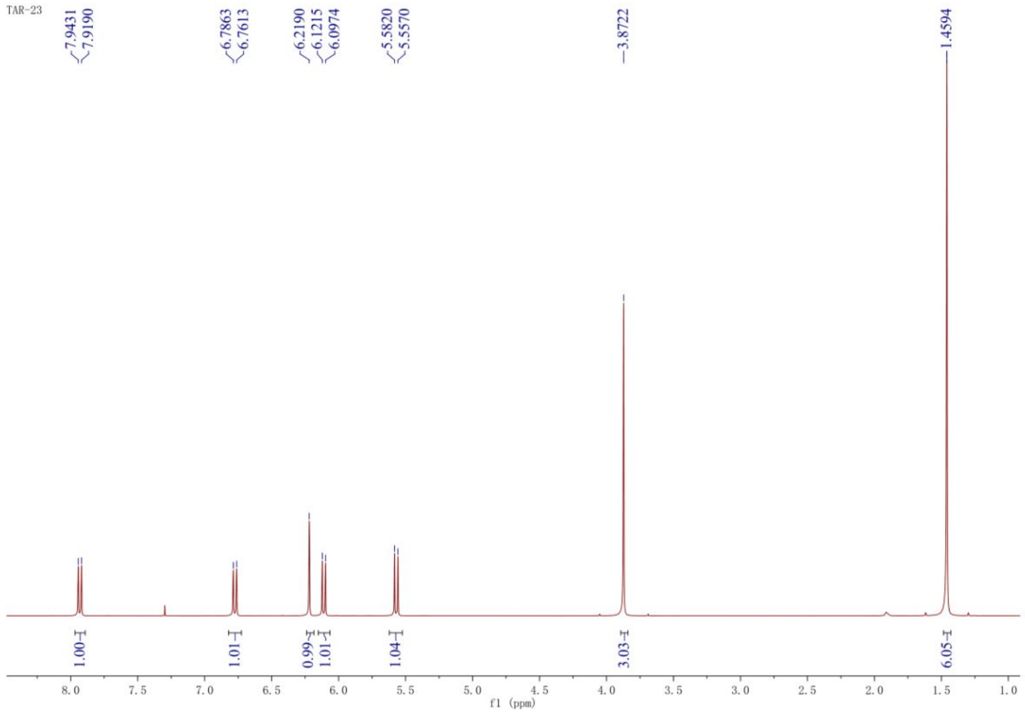


**Figure S1.** $^1$H NMR spectrum of compound 5-MOS in $CDCl_3$.

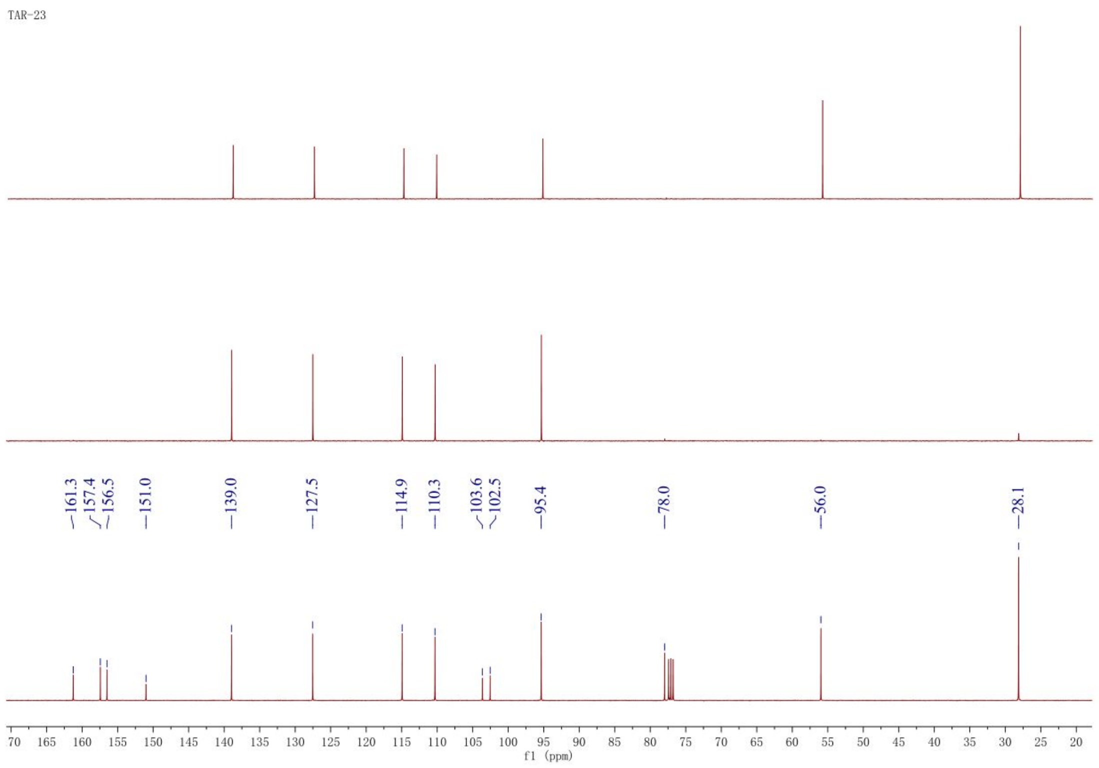


**Figure S2.** $^{13}C$ NMR and DEPT spectra of compound 5-MOS in $CDCl_3$.

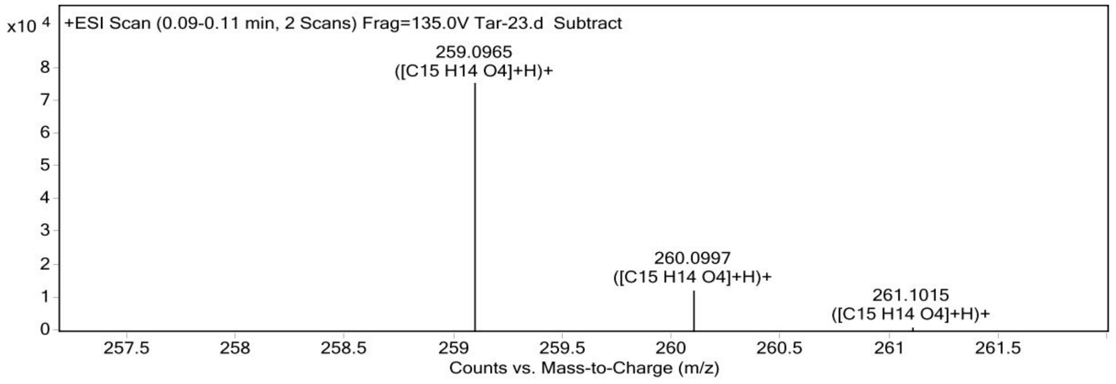


**Figure S3.** HRMS spectrum of compound 5-MOS.

**Table S1.** Crystallographic and structural refinement data of 5-MOS.[a]

| | |
|---|---|
| Empirical formula | $C_{15}H_{14}O_4$ |
| Formula weight | 258.26 |
| Temperature (K) | 301(2) |
| Wavelength (Å) | 1.54178 |
| Crystal system | Monoclinic |
| space group | C 2/m |
| a (Å) | 17.9080(4) |
| b (Å) | 6.8267(2) |
| c (Å) | 12.7633(3) |
| α (°) | 90 |
| β (°) | 124.5640(10) |
| γ (°) | 90 |
| Volume (Å3) | 1284.93(6) |
| Z | 4 |
| θ range (°) | 7.046 to 68.344 |
| Index ranges | -20<=h<=21, -7<=k<=8, -15<=l<=14 |

[a]Crystallographic data for the structures reported in this paper have been deposited with the Cambridge Crystallographic Data Centre as supplementary publication no. CCDC: 2154427 for 5-MOS.

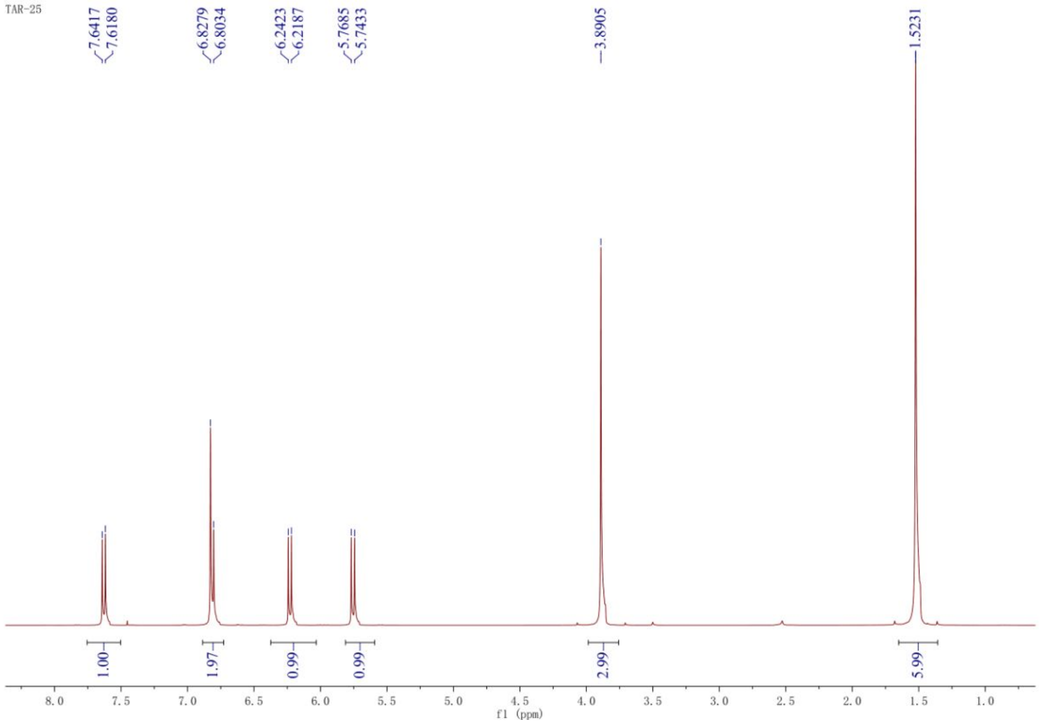


**Figure S4.** $^{1}$H NMR spectrum of compound 6-MOS in $CDCl_3$.

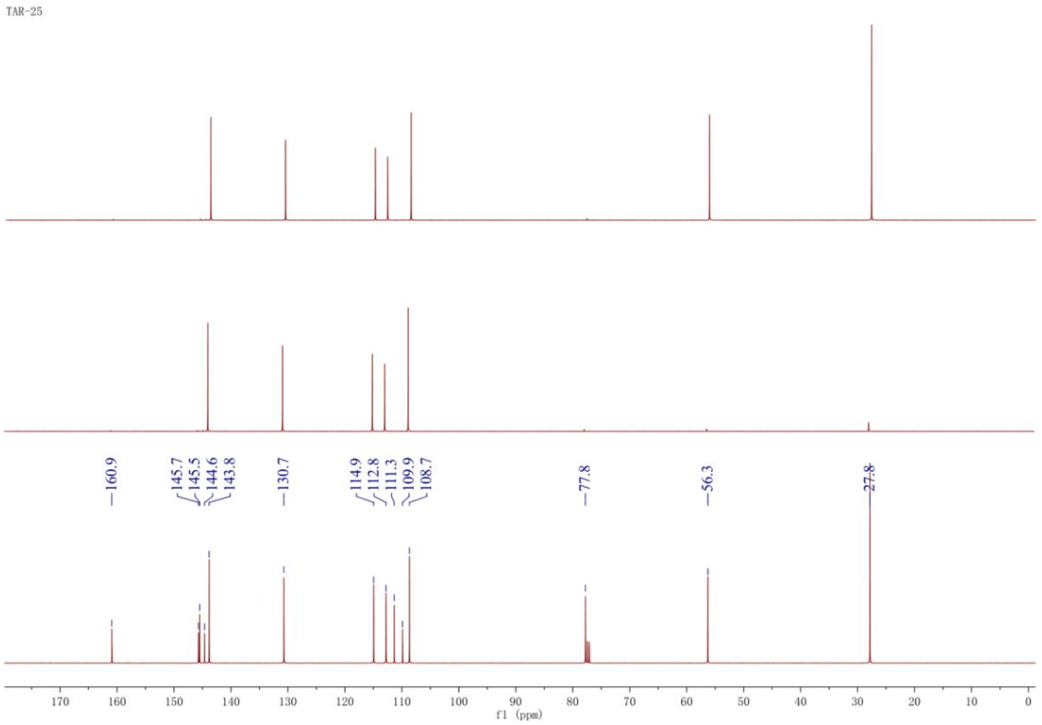


**Figure S5.** $^{13}$C NMR and DEPT spectra of compound 6-MOS in $CDCl_3$.

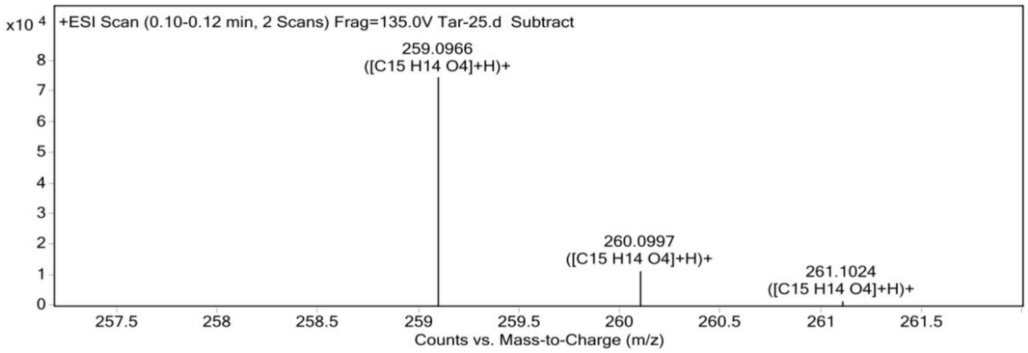


**Figure S6.** HRMS spectrum of compound 6-MOS.

**Table S2.** Crystallographic and structural refinement data of 6-MOS.[a]

| | |
|---|---|
| Empirical formula | $C_{15}H_{14}O_4$ |
| Formula weight | 258.26 |
| Temperature (K) | 302(2) |
| Wavelength (Å) | 1.54178 |
| Crystal system | Orthorhombic |
| space group | Pccn |
| a (Å) | 12.7209(2) |
| b (Å) | 23.9032(4) |
| c (Å) | 8.47540(10) |
| α (°) | 90 |
| β (°) | 90 |
| γ (°) | 90 |
| Volume (Å3) | 2577.12(7) |
| Z | 8 |
| θ range (°) | 3.936 to 68.332 |
| Index ranges | -15<=h<=15, -28<=k<=28, -10<=l<=9 |

[a]Crystallographic data for the structures reported in this paper have been deposited with the Cambridge Crystallographic Data Centre as supplementary publication no. CCDC: 2154530 for 6-MOS.

**Table S3.** Optical properties of 5-MOS and 6-MOS.

| | | $\lambda_{abs}$ | $\lambda_{em}$ | $\Phi$ (%)[b] | $\tau$ (ns)[c] | $k_r$(×$10^7$ $s^{-1}$ ) | $k_{nr}$(×$10^7$ $s^{-1}$) |
|---|---|---|---|---|---|---|---|
| | soln | 328 | 473 | 11.8 | 3.09 | 3.8 | 28.6 |
| 5-MOS | aggr | 328 | 511 | 2.3 | 0.65 | 3.5 | 149.7 |
| | solid | —— | 470 | 18.5 | 5.53 | 3.3 | 14.8 |
| | soln | 352 | 428 | 2.1 | 0.62 | 3.4 | 158.6 |
| 6-MOS | aggr | 352 | 482 | 9.9 | 1.59 | 6.2 | 56.6 |
| | solid | —— | 452 | 6.5 | 3.13 | 2.1 | 29.9 |

$\tau$: average fluorescence lifetime calculated by $\tau = \Sigma(Ai\tau_i)^2/\Sigma Ai\tau_i$, where $A_i$ is the pre-exponential for lifetime $\tau_i$. $\Phi$ = fluorescence quantum yield measured by using an integrating sphere. $k_r$ = radiative decay rate ($\Phi/\tau$). $k_{nr}$ = nonradiative decay rate $(1-\Phi)/\tau$. soln = DMSO solution (10 μM), aggr = mixed solution (DMSO/water=1/99).

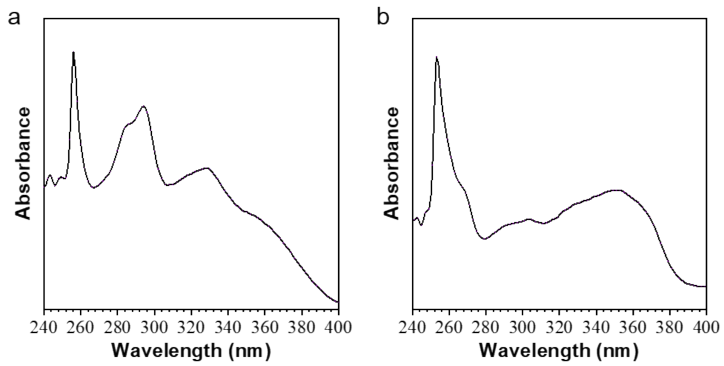


**Figure S7.** UV-vis absorption spectra of 5-MOS (a) and 6-MOS (b) in DMSO solution. Concentration: 10 μM.

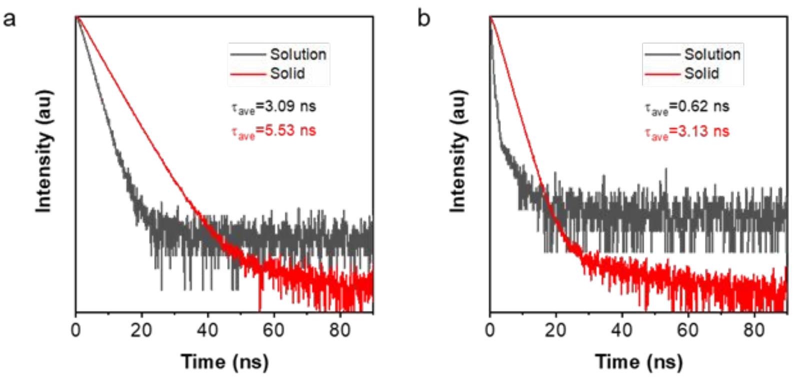


**Figure S8.** The transient PL decay curves of 5-MOS (a) and 6-MOS (b) with different state. Excitation wavelength: 340 nm.

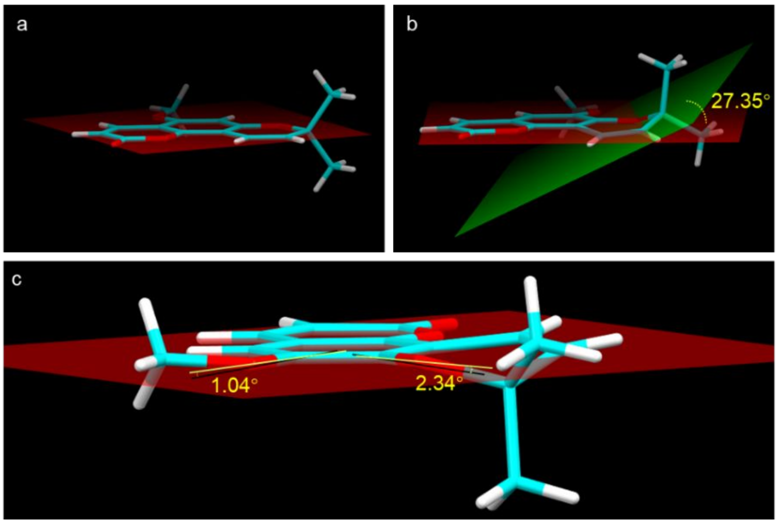


**Figure S9.** Crystal structures and dihedral angle and torsion angle of 5-MOS (a) and 6-MOS (b–c).

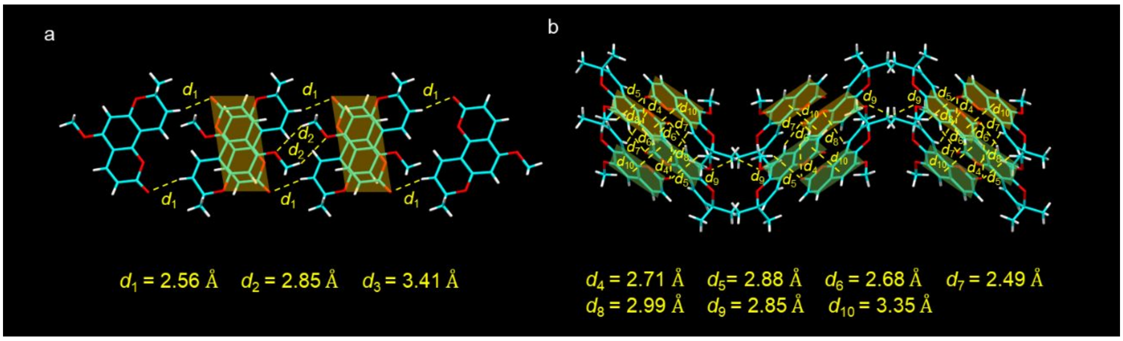


**Figure S10.** (a) Single crystal packing structure and intermolecular noncovalent interactions of 5-MOS. (b) Single crystal packing structure and intermolecular noncovalent interactions of 6-MOS.

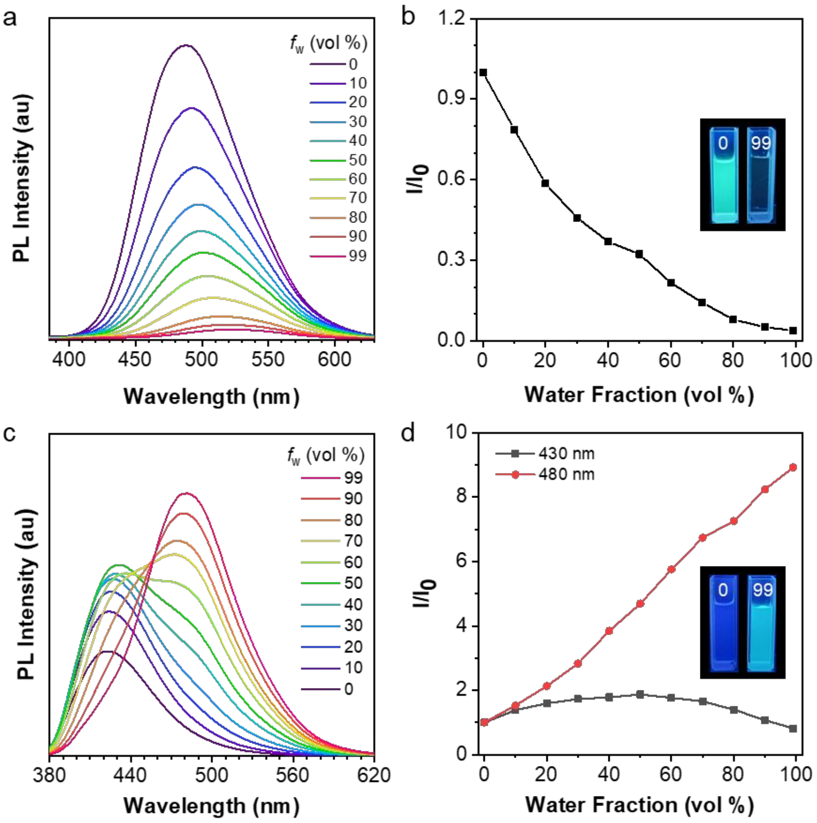


**Figure S11.** (a) PL spectra of 5-MOS in ethanol/water mixtures with different $f_w$. (b) Plots of the $I/I_0$ versus $f_w$ of 5-MOS, where $I$ and $I_0$ are the maximal PL intensity in ethanol/water mixtures with different $f_w$ and in ethanol solution, respectively. Inset: fluorescence images of 5-MOS at $f_w$= 0% and $f_w$= 99% taken under 365 nm UV light irradiation. (c) PL spectra of 6-MOS in ethanol/water mixtures with different $f_w$. (d) Plots of $I/I_0$ versus $f_w$ of 6-MOS at 430 nm and 480 nm. Inset: fluorescence images of 6-MOS at $f_w$= 0% and $f_w$= 99% taken under 365 nm UV light irradiation. Concentration: 10 µM. Excitation wavelength: 328 nm for 5-MOS and 352 nm for 6-MOS.

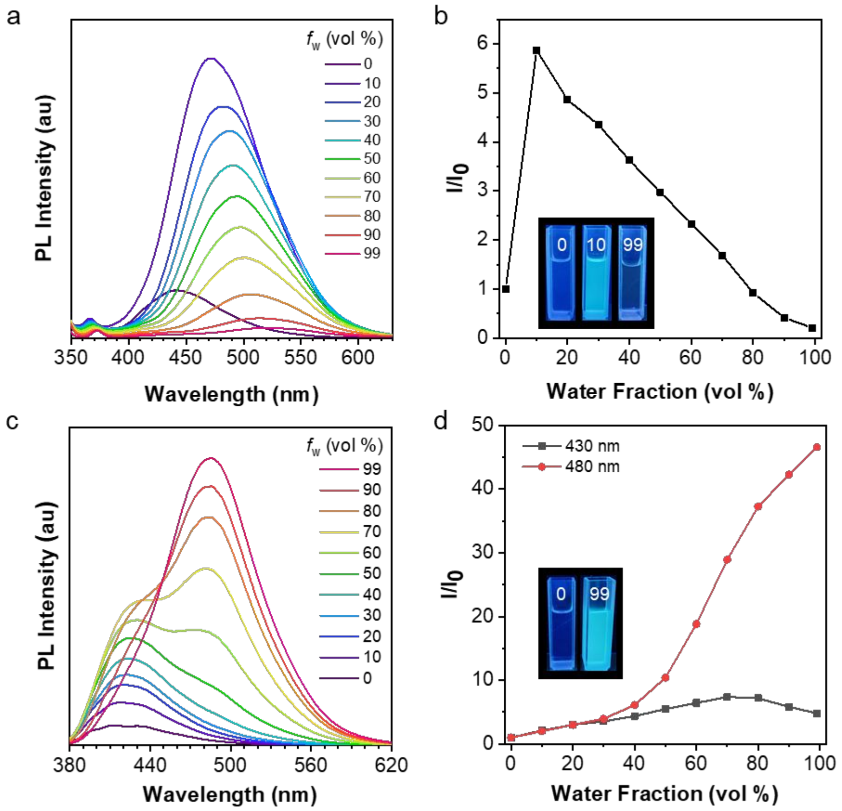


**Figure S12.** (a) PL spectra of 5-MOS in THF/water mixtures with different $f_w$. (b) Plots of the $I/I_0$ versus $f_w$ of 5-MOS, where $I$ and $I_0$ are the maximal PL intensity in THF/water mixtures with different $f_w$ and in THF solution, respectively. Inset: fluorescence images of 5-MOS at $f_w$ = 0% and $f_w$ = 99% taken under 365 nm UV light irradiation. (c) PL spectra of 6-MOS in THF/water mixtures with different $f_w$. (d) Plots of $I/I_0$ versus $f_w$ of 6-MOS at 430 nm and 480 nm. Inset: fluorescence images of 6-MOS at $f_w$ = 0% and $f_w$ = 99% taken under 365 nm UV light irradiation. Concentration: 10 μM. Excitation wavelength: 328 nm for 5-MOS and 352 nm for 6-MOS.

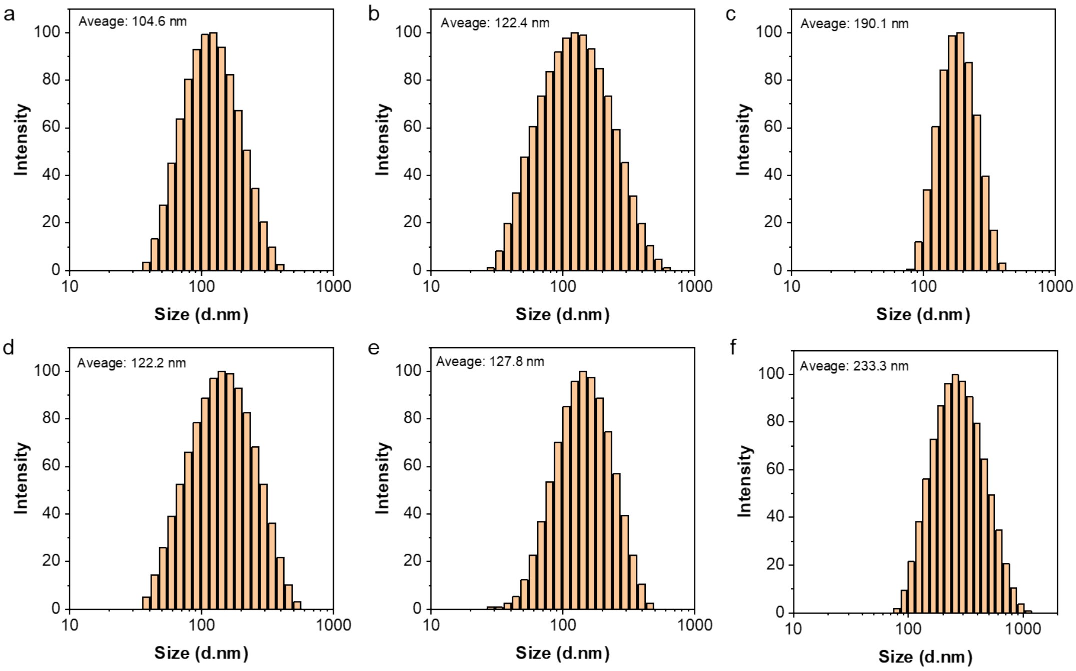


**Figure S13.** DLS result of 5-MOS in a DMSO/water mixture (a), ethanol/water mixture (b), THF/water mixture (c). DLS result of 6-MOS in a DMSO/water mixture (d), ethanol/water mixture (e), THF/water mixture (f). Concentration: 10 μM. $f_w$ =99%.

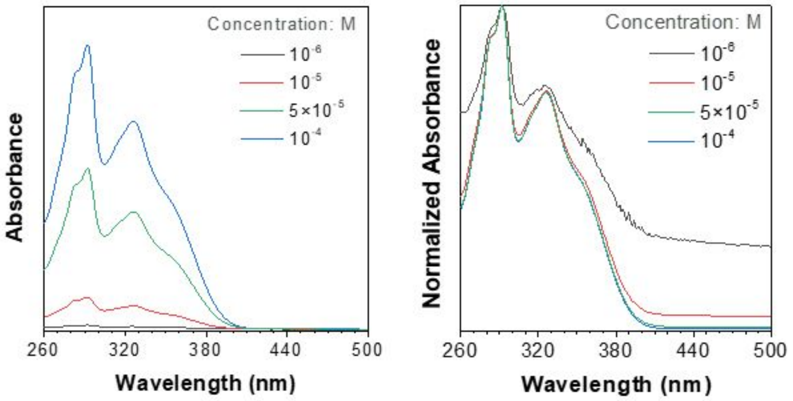


**Figure S14.** UV-vis absorption spectra of 5-MOS at different concentrations in ACN.

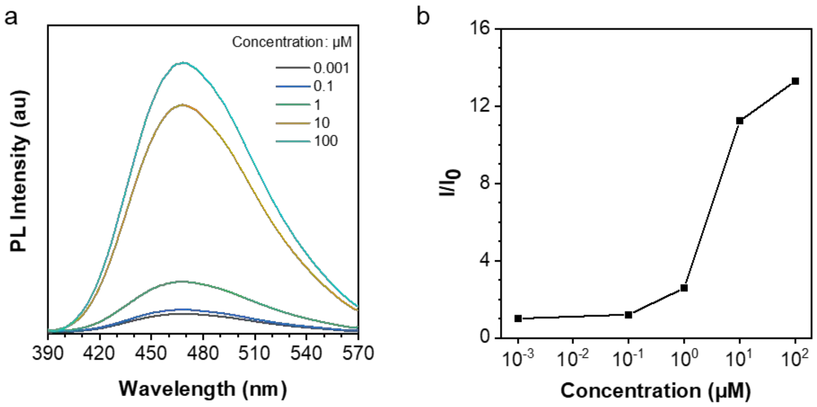


**Figure S15.** (a) PL spectra of 5-MOS in ACN at different concentrations. (b) Plots of the relative emission intensity ($I/I_0$) versus concentration of 5-MOS, where $I_0$ and $I$ are the maximal PL intensity in ACN at 0.001 μM, and other different concentration, respectively. Excitation wavelength: 328 nm.

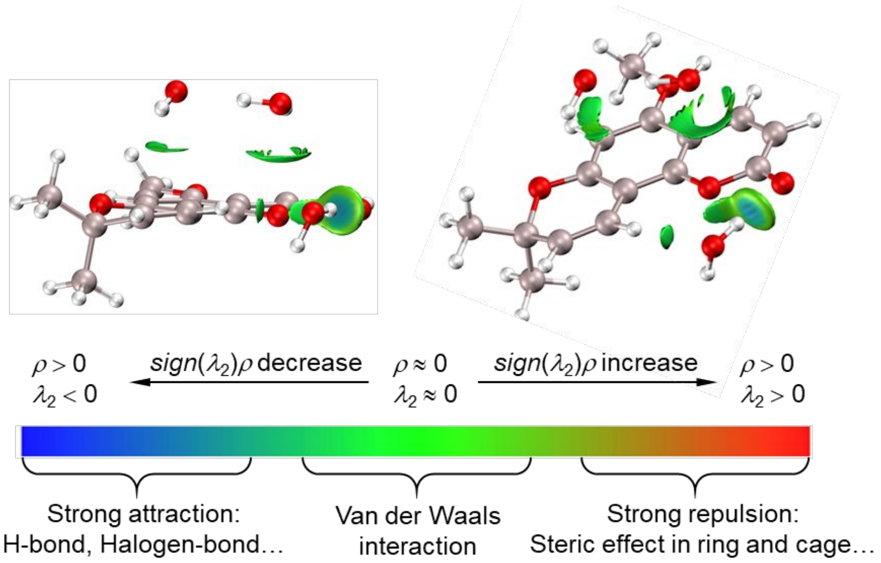


**Figure S16.** Intermolecular interaction between 5-MOS and water molecules in ground state calculated via Independent gradient model based on Hirshfeld partition (IGMH). δg_inter isovalue = 0.0005.

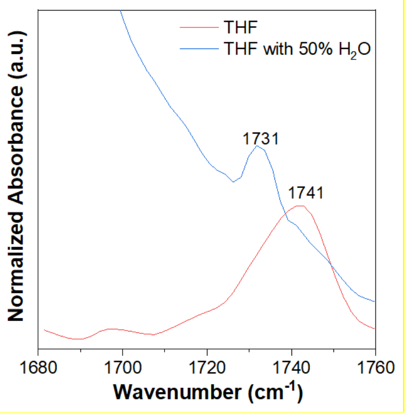


**Figure S17.** IR spectra of 5-MOS showing the carbonyl frequencies in dry THF and THF/water (50:50 v/v) mixture. Concentration: 2.5 mg/mL .

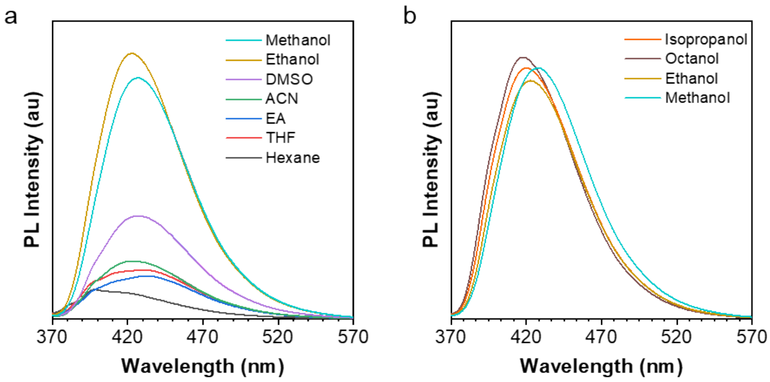


**Figure S18.** (a) PL spectra of 6-MOS in the solvents with different polarity. (b) PL spectra of 6-MOS in different protic solvents. Concentration: 10 µM.

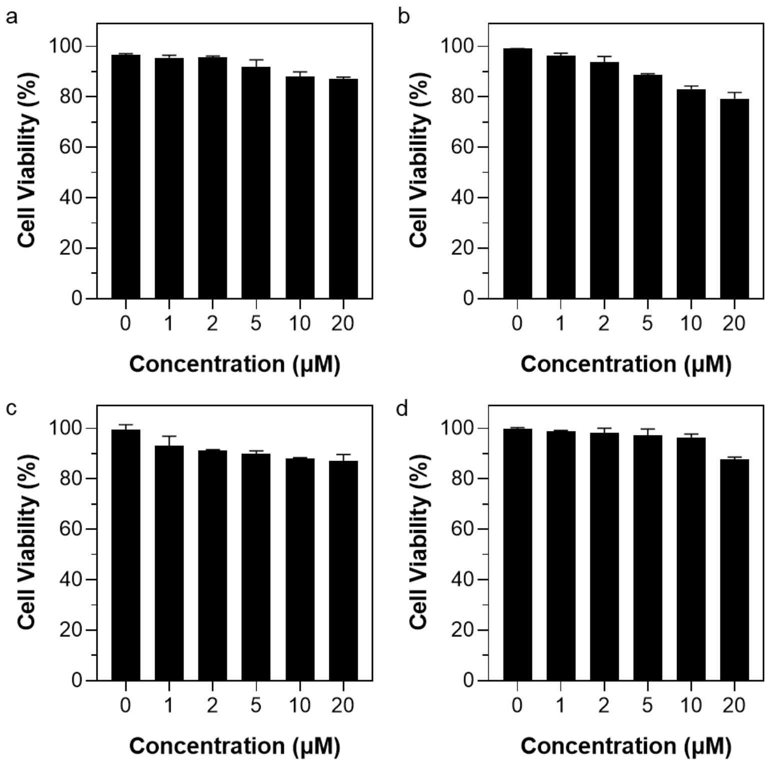


**Figure S19.** Cell viability of MHCC97H (a), HEL-1 (b) cells incubated with 5-MOS. Cell viability of MHCC97H (c), HEL-1 (d) cells incubated with 6-MOS.

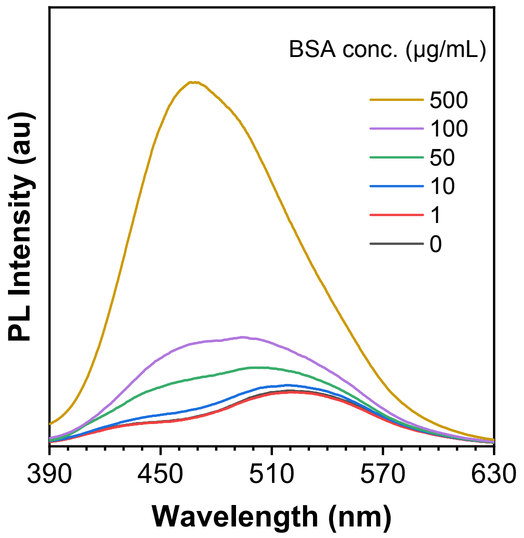


**Figure S20.** PL spectra of 5-MOS in bovine serum albumin (BSA) aqueous solutions with different concentrations. Excitation wavelength: 328 nm.

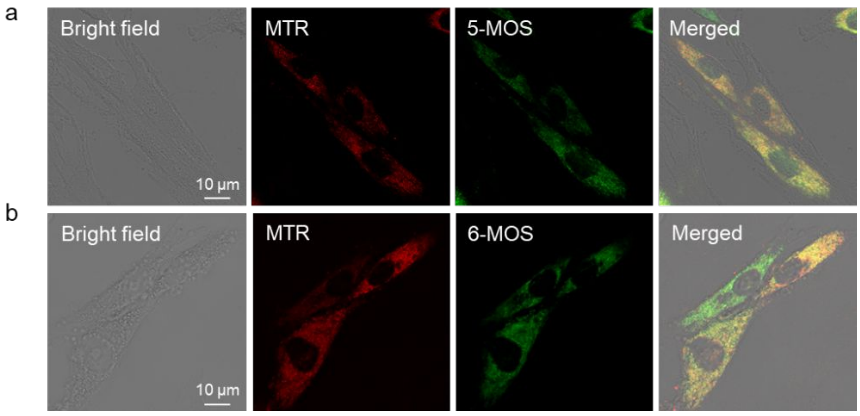


**Figure S21.** (a) Confocal images of HEL-1 cells stained with MTR for 15 min and 5-MOS for 15 min. (b) Confocal images of HEL-1 cells stained with MTR for 15 min and 6-MOS for 1.5 h. Concentration of MTR: 50 nM. Concentration of 5-MOS and 6-MOS: 10 µM. Excitation wavelength: 405 nm for 5-MOS and 6-MOS, and 561 nm for MTR.

## Reference

1. Lu, T, Chen, Q. Independent gradient model based on Hirshfeld partition: A new method for visual study of interactions in chemical systems. *J Comput Chem* 2022; **43**(8): 539-55.
2. Grimme, S, Ehrlich, S, Goerigk, L. Effect of the Damping Function in Dispersion Corrected Density Functional Theory. *J Comput Chem* 2011; **32**(7): 1456-65.
